# Supplementary material for: Analysis of Common SNPs across Continents Reveals Major Genomic Differences between Human Populations
Source: Genes (Basel). 2022 Aug 18;13(8):1472. doi: 10.3390/genes13081472 (PMC9408407; doi:10.3390/genes13081472)
Supplement: Supplementary file 1 [file genes-13-01472-s001.zip › genes-1766591-supplementary/ATLAS3protocols.pdf]

March 15, 2022

SUMMARY ON OCE-specific SNP dataset. (the data are in Moscow BIO Linux workstation in /home/afedorov/NOV2021)

1) Start from Simons' dataset. The program `OceaniaSimonStep1.pl` counts number of Alt-allele SNPs in OCE: /Aus|Bou|Pap/, which should be 7 or more alleles (among 19 people) The program also counts this allele in OCE-flanking populations: /Dus|Mao|Igo|Hav/. In the rest of Simons' populations this allele must be absent. It creates the phase-1 OCE-specific SNPs (OCE_SNPs_*$chr*), where *$chr* means the number of chromosome (from 1 to 22)

2) Comparison with 1000G dataset. The second program `Oceania1000gStep2.pl` compares phase-1 OCE-specific SNPs with all 2504 individuals from 1000G. The number of "OCE-specific phase-1" alleles in all 1000G should be less or equal to the number of these alleles among 19 OCE people. Thus, the frequency of OCE-specific SNPs at this second step must be >100 times frequent than in the rest of the world. This computation creates phase-2 OCE-specific SNPs, which are stored in the OceaniaSpecificSNPs_*$chr* files. After application of V3 (OCE freq in 1000G unknown!!, yet total freq is <150x than in Oceania) in `OceaniaSpecificV3_$chr` files I got **71,848** SNPs.

3) Final comparison with Estonian dataset using `OceanEstoniaStep3.pl` program. The number of OCE-specific alleles among all Estonian DB people must be 4 or less. This is the final phase-3 purification (very weak with main purpose to print populations that have Region-specific alleles in the final table). The main results are in the files specific for each chromosome: OceaniaSpecificSNPsEstonia2_*$chr* and also the combined file for all chromosomes: `OceaniaSpecificSNPsEstonia2_ALL`. This file contains the number of OCE-specific alleles in all datasets (Simons, 1000G, and Estonian) and also the name of populations, where they present. See this table below,

Col-1	Col-2.	Col-3.	Col-4	Col-5.	Col-6	Col-7	Col-8	Col9.	Col10.	Col11.	Col12.	Col13	Col14	Col15	Col16	Col-17+				
rs373000721	9	0	0	0	CHR1	833529	rs373000721	G	A	0	0	0	0	0	NOT	Koi_OCE	Kos_OCE	Koi_OCE		
rs367861531	10	0	0	0	CHR1	835831	rs367861531	G	A	0	0	0	1	0	YRI		Koi_OCE	Kos_OCE	Koi_OCE	
rs369581566	10	0	0	0	CHR1	837238	rs369581566	G	A	0	0	0	0	0	NOT	Koi_OCE	Kos_OCE	Koi_OCE		
rs377052638	8	0	0	0	CHR1	837992	rs377052638	C	G	0	0	0	0	0	NOT	Koi_OCE	Kos_OCE	Koi_OCE		
rs373451994	10	0	0	0	CHR1	839636	rs373451994	G	A	0	0	0	0	0	NOT	Koi_OCE	Kos_OCE	Koi_OCE		
rs146441147	9	0	0	0	CHR1	979559	rs146441147	C	T	0	0	0	0	0	NOT	Kos_OCE	Koi_OCE			
rs372205897	12	0	0	0	CHR1	1029701	rs372205897	C	T	0	0	0	0	0	NOT	Kos_OCE	Koi_OCE			
rs373322116	12	0	0	0	CHR1	1047802	rs373322116	G	A	0	0	0	0	0	NOT	Kos_OCE	Koi_OCE			
rs375647989	8	0	0	0	CHR1	1107859	rs375647989	A	G	0	0	0	0	0	NOT	Kos_OCE	Kos_OCE			
rs371814109	8	0	0	0	CHR1	1109872	rs371814109	A	G	0	0	0	0	0	NOT	Kos_OCE	Kos_OCE			
rs373310048	8	0	0	0	CHR1	1110271	rs373310048	G	A	0	0	0	0	0	NOT	Kos_OCE	Kos_OCE			
rs11811026	8	0	0	0	CHR1	1112124	rs11811026	C	A	0	0	0	0	0	ASW		Kos_OCE	Kos_OCE		
rs11811377	8	0	0	0	CHR1	1112318	rs11811377	C	A	1	0	0	1	0	CLM	YRI	ASW		Kos_OCE	Kos_OCE
rs377508467	8	0	0	0	CHR1	1112457	rs377508467	T	G	0	0	0	0	0	NOT	Kos_OCE	Kos_OCE			
rs375453018	8	0	0	0	CHR1	1114523	rs375453018	C	T	0	0	0	1	0	GWD	ASW		Kos_OCE	Kos_OCE	
rs374835744	8	0	0	0	CHR1	1114552	rs374835744	C	T	0	0	0	1	0	GWD	ASW		Kos_OCE	Kos_OCE	
rs368714026	7	0	0	0	CHR1	1116266	rs368714026	C	T	0	0	0	0	0	NOT	Kos_OCE	Kos_OCE			
s1x1118971	7	0	0	0	CHR1	1118971	s1x1118971	C	G	0	0	0	0	0	NOT	Baj_OCE	Koi_OCE	Kos_OCE	Koi_OCE	
rs551036611	12	0	0	0	CHR1	1302130	s1x1302130	G	A	0	0	2	0	0	KHV		Baj_OCE	Agt_OCE		
s1x1344958	7	0	0	0	CHR1	1344958	s1x1344958	A	G	0	0	0	0	0	NOT	Baj_OCE	Agt_OCE			
rs369955862	7	0	0	0	CHR1	1381934	rs369955862	A	G	0	0	0	0	0	NOT	Leb_OCE	Baj_OCE	Agt_OCE		
s1x1385293	7	0	0	0	CHR1	1385293	s1x1385293	G	A	0	0	0	0	0	NOT	Baj_OCE	Agt_OCE			
rs367639744	10	0	0	0	CHR1	1471892	rs367639744	C	T	0	0	0	0	0	NOT	Koi_OCE	Koi_OCE			
s1x1503254	7	0	0	0	CHR1	1503254	s1x1503254	T	C	0	0	0	0	0	NOT	Agt_OCE				

Col-1 (rs-ID if existed in 1000G, if not then our ID: s1x1503254, where first "s" means Simons project, following the chr number, then 'x' separator and position on chr)
Col-2 Number of OCE-specific alleles counts in phase-1 in 19 OCE people from /Aus|Bou|Pap/

```
Col-3 Number of OCE-specific alleles counts in phase-1 in 19 neighboring OCE people from /Dus|Mao|Igo|Hav/
Col-4 Always 0, because this item was skipped from AMR-specific SNPs
Col-5 Always 0, because this item was skipped from AMR-specific SNPs
Col-6 Chromosome number
Col-7 Position on chr
Col-8 repetition of column-1
Col-9 Reference allele
Col-10 Alternative allele
Col-11 Number of OCE-specific alleles in all AMR from 1000G
Col-12 Number of OCE-specific alleles in all EUR from 1000G
Col-13 Number of OCE-specific alleles in all EAS from 1000G
Col-14 Number of OCE-specific alleles in all AFR from 1000G
Col-15 Number of OCE-specific alleles in all SAS from 1000G
Col-16 All populations from 1000G were the OCE-specific allele was observed. If not observed the NOT will appeared
Col-17+ All populations from ESTONIAN DB were the OCE-specific allele was observed. For example, Kos_OCE
```

SUMMARY ON AMR-specific SNP dataset. (the data are in Moscow BIO Linux workstation in /home/afedorov/NOV2021)

1) Start from Simons' dataset. The program `AmericanSNPs2020.pl` counts number of Alt-allele SNPs in AMR pure tribes: /Cha|Kar|Sur|Pia/, which should be 3 or more alleles (among 8 people) [COLUMN-2]. The program also counts this allele in AMR more diverse populations, that have admixture: /May|Mix|Pim|Que|Zap/ 14 people (admixed with 30% Europeans and Africans genes) [COLUMN-3]. In order to qualify as AMR-specific SNP I created an additional requirement for the counts: Col2 + Col3 >= 7 (program `purgeNativeAmericanSNPs.pl`). Finally, I also counted the number of alleles in the AMR-flanking populations from Arctic (ARC), that little intersect with AMR: /Ale|Chu|Esk|Ite|Tli|Ulc/ [COLUMN-4] and also Siberia (SIB): /Eve|Man|Yak|Tub/ [COLUMN-5]. In the rest of Simons' populations this allele must be absent. It creates the phase-1 AMR-specific SNPs (AMR_SNPs_*$chr*), where *$chr* means the number of chromosome (from 1 to 22)

2) Comparison with 1000G dataset. The second program `America1000gStep2_v2.pl` compares phase-1 AMR-specific SNPs with all 2504 individuals from 1000G. The number of "AMR-specific phase-1" alleles in all 1000G dataset is divided by 5 regions (EAS, SAS, EUR, AFR, AMR). The number of AMR-specific alleles in AMR populations should be more than the number of these alleles in the rest four regions multiplied by 10 [e.g. (AFR+EAS+SAS+EUR)*10] . Thus, the frequency of AMR-specific SNPs at this second step in AMR must be >40 times frequent than in the rest of the world. Since AMR populations from 1000G are very admixed with European and African genomes, this requirement equivalents that in pure AMR-populations the frequency of these alleles should be >100x than their frequency in the rest of the World. This computation creates phase-2 AMR-specific SNPs, which are stored in the AmericaSpecificSNPs_*$chr* files. After application of V3 (AMR freq in 1000G >=9% and >18% which is taking into account admixture AMR with other continents) in `AmericaSpecificV3_`*`$chr`* files I got **4,133** SNPs.

3) Final comparison with Estonian dataset using `AmericanSNPsESTONIA.pl` program. The number of AMR-specific alleles among all Estonian DB people must be 4 or less. [A vast majority of Estonian DB are not American populations. They will be listed in the final table]. This is the final phase-3 purification (very weak with main purpose to print populations that have Region-specific alleles in the final table). The main results are in the files specific for each chromosome: `AmericaSpecificSNPsEstonia2_`*`$chr`* and also the combined file for all chromosomes: `AmericaSpecificSNPsEstonia2_ALL`. This file contains the number of AMR-specific alleles in all datasets (Simons, 1000G, and Estonian) and also the name of populations, where they present. See example of this table for OCE-specific alleles above.

SUMMARY ON EAS-specific (China-Specific) SNP dataset. (the data are in Moscow BIO Linux workstation in /home/afedorov/NOV2021)

4) Start from Simons' dataset. The program `ChinaSimonStep1.pl` counts number of Alt-allele SNPs in EAS pure populations: /Dai|Han|Jap|Kor|Mia|Nax|She|Tuj|YiX/, which should be 8 or more alleles (among 22 people) [COLUMN-2]. The program also counts this allele in EAS more diverse populations, that have admixture: /Cam|TuX|Lah|Xib|Hez|Oro|Mon|Dau|Bur|Ami|Ata|Kin/ [COLUMN-3]. Finally, I also counted the number of alleles in the EAS-flanking populations from the south, that little intersect with EAS: /Kus|BUm|Tam|Leb|Luz|Ulc|Mur|Igo|Haw|Dus/ [COLUMN-4] and also Central Asia: /Eve|EVk|Yak|Tub|Sho|Sak|Alt|Kal|Kaz|Kyr|Uyg/ [COLUMN-5]. In the rest of Simons' populations this allele must have 4 or less counts. It creates the phase-1 China-specific SNPs (CHI_SNPs_*$chr*), where *$chr* means the number of chromosome (from 1 to 22)

5) Comparison with 1000G dataset. The second program `China1000gStep2_v2.pl` compares phase-1 EAS-specific SNPs with all 2504 individuals from 1000G. The number of "EAS-specific phase-1" alleles in all 1000G dataset is divided by 5 regions (EAS, SAS, EUR, AFR, AMR). The number of EAS-specific alleles in EAS populations should be more than the number of these alleles in the rest four regions multiplied by 10 [e.g. (AFR+AMR+SAS+EUR)*10] . Thus, the frequency of AMR-specific SNPs at this second step in AMR must be >40 times frequent than in the rest of the world. This restriction is the lower than for AMR and OCE, because we have 10 times less EAS-specific SNPs. This computation creates phase-2 EAS-specific SNPs, which are stored in the ChinaSpecificSNPs_*$chr* files. After application of V3 (EAS freq in 1000G >=18%) in `ChinaSpecificV3_`*$chr* files I got **441** SNPs.

6) Final comparison with Estonian dataset using `ChinaEstoniaStep3.pl` program. The number of EAS-specific alleles among all Estonian DB people must be 4 or less. [A vast majority of Estonian DB are not EAS populations. They will be listed in the final table]. This is the final phase-3 purification (very weak with main purpose to print populations that have Region-specific alleles in the final table). The main results are in the files specific for each chromosome: `ChinaSpecificSNPsEstonia2_$`*chr* and also the combined file for all chromosomes: `ChinaSpecificSNPsEstonia2_ALL`. This file contains the number of EAS-specific alleles in all datasets (Simons, 1000G, and Estonian) and also the name of populations, where they present. See example of this table for OCE-specific alleles above.

SUMMARY ON AFR-specific SNP dataset. (the data are in Moscow BIO Linux workstation in /home/afedorov/NOV2021)

1) Start from Simons' dataset. The program `AfricaSimonStep1.pl` counts number of Alt-allele SNPs in AFR pure populations: /Moz|Ban|Bia|Mbu|Gam|Luo|Mas|Luh|Som|JuX|Yor|Esa|Man|Men|Kho|Din|Sah/, which should be 17 or more alleles (among 47 people) [COLUMN-2]. The program DOES NOT count this allele in AFR diverse populations, thus, [COLUMN-3, 4, and 5] are empty (0). In the rest of Simons'

populations this allele must have 2 or less counts. It creates the phase-1 AFR-specific SNPs (CHI_SNPs_*$chr*), where *$chr* means the number of chromosome (from 1 to 22)

2) Comparison with 1000G dataset. The second program `Africa1000gStep2_v2.pl` compares phase-1 AFR-specific SNPs with all 2504 individuals from 1000G. The number of “AFR-specific phase-1” alleles in all 1000G dataset is divided by 5 regions (EAS, SAS, EUR, AFR, AMR). The number of AFR-specific alleles in AFR populations should be more than the number of these alleles in the rest four regions multiplied by 10 [e.g. (EAS+AMR+SAS+EUR)*10] . Thus, the frequency of AFR-specific SNPs at this second step in AFR must be >40 times frequent than in the rest of the world. This restriction is the lower than for AMR and OCE, because we have strong African admixture in AMR and Southern Europe. This computation creates phase-2 AFR-specific SNPs, which are stored in the AfricanSpecificSNPs_*$chr* files. After application of V3 (AFR freq in 1000G >=18%) in `AfricaSpecificV3_$chr` files I got **112,658** SNPs.

3) Final comparison with Estonian dataset using `AfricanSNPsESTONIA.pl` program. The number of AFR-specific alleles among all Estonian DB people must be 4 or less. [A vast majority of Estonian DB are not AFR populations. They will be listed in the final table]. This is the final phase-3 purification (very weak with main purpose to print populations that have Region-specific alleles in the final table). The main results are in the files specific for each chromosome: `AfricaSpecificSNPsEstonia2_$chr` and also the combined file for all chromosomes: `AfricaSpecificSNPsEstonia2_ALL`. This file contains the number of AFR-specific alleles in all datasets (Simons, 1000G, and Estonian) and also the name of populations, where they present. See example of this table for OCE-specific alleles above.

SUMMARY ON EUR-specific SNP dataset. (the data are in Moscow BIO Linux workstation in /home/afedorov/NOV2021)

1) Start from Simons’ dataset. The program `EuropeSimonStep1.pl` counts number of Alt-allele SNPs in EUR pure populations: /Alb|Bas|Ber|Bul|Cre|Eng|Est|Fin|Fre|Gre|Hun|Ice|Nor|Orc|Pol|Rus|Saa|Sar|Spa|Tus/, which should be 15 or more alleles (among 41 people) [COLUMN-2]. The program counts these alleles in NON-EUR diverse populations, which should be less than two counts in total. These NON-EUR include the following: /Dai|Han|Jap|Kor|Mia|Nax|She|Tuj|YiX|Moz|Ban|Bia|Mbu|Gam|Luo|Mas|Luh|Som|JuX|Yor|Esa|Man|Men|Kho|Din|Sah/, which are EAS and AFR-populations[COLUMN- 5]. And also /Cam|TuX|Lah|Xib|Hez|Oro|Mon|Dau|Bur|Ami|Ata|Kin/and /Kus|BUm|Tam|Leb|Luz|Ulc|Mur|Igo|Haw|Dus/ which are neighboring to EAS populations. In the rest of Simons’ populations this allele must have only one or zero counts. It creates the phase-1 EUR-specific SNPs (EUR_SNPs_*$chr*), where *$chr* means the number of chromosome (from 1 to 22). ALTOGETHER I GOT **6585** SNPs.
2) Comparison with 1000G dataset. The second program `Europe1000gStep2_v2.pl` compares phase-1 EUR-specific SNPs with all 2504 individuals from 1000G. The number of “EUR-specific phase-1” alleles in all 1000G dataset is divided by 5 regions (EAS, SAS, EUR, AFR, AMR). The number of EUR-specific alleles in EUR populations should be more than the number of these alleles in AFR plus EAS multiplied by 20 plus number of this allele in SAS population ($afr+$asia)*20 + $ind*1 . In other words, this EUR-specifi allele must be 40x less frequent in AFR and EAS and also less frequent than in SAS. Thus, the frequency of AFR-specific SNPs at this second step in AFR must be >40 times frequent than in the rest of the world. This restriction is chosen

because we have strong European admixture in AMR and India (SAS). This computation creates phase-2 EUR-specific SNPs, which are stored in the EuropeSpecificSNPs_*$chr* files. ALTOGETHER I GOT **3925** SNPs.
After application of V3 (EUR freq in 1000G >=18%) in `EuroSpecificV3_$chr` files I got **1539** SNPs.
After application of V3 (EUR freq in 1000G >=15%) in `EuroSpecificV3_$chr` files I got **2484** SNPs.

3)

March 25, 2022

New thresholds for Region-Specific SNPs.

Table S1. Distribution the numbers of common region-specific SNPs, which MAF > 18%.

Region	Step-1. Number of region-specific SNPs in Simons' Database	Step-2. Number of region-specific SNPs purged on 1000 Genomes	Step-3. Number of region-specific SNPs purged on EGDP DB	Step-4. Number of clusters of Region-specific SNPs
Africa	204,983	112,658	77,820	28,774
Americas	46,994	4,133	3,348	3,222
East Asia	7,789	441	362	272
Europe	6,585	2,484	1,911	1,394
Oceania	77,437	*71,848 **	1,358	453

Step-1: Processing SNPs from Simons' Project. Characterization and counting the number of common region-specific SNPs (MAF >18%), which frequency in other regions (those without noticeable admixture).

Step-2. Characterization the number of common region-specific SNPs from Step-1, which MAF in the same region is also >18% in the 1000 Genomes DB. Verification that the frequencies of these region-specific SNP alleles in 1000 Genomes are also reduced at least 40 times in other regions.

*Note that Oceania populations are absent in 1000 Genomes, thus, the requirement for MAF >= 18% is omitted for OCE at Step-2.
However, this requirement (MAF>18%) is enforced in the next Step-3 for the EGDP Database, which has 51 individuals from OCE populations.

Step-3. Characterization the number of common region-specific SNPs from Step-2, which MAF is also significant (>16%) in EGPD Database in the same region. Confirmation that frequencies of these region-specific SNP alleles in EGPD are drastically reduced in other regions.

Step-4. Grouping of neighboring region-specific SNPs from Step-3 into clusters when the distance between neighboring SNPs is less than 5 Kb.

Final results are in the files for each autosome (1..22): **FinalRegion_chr**

```
afedorov@bio:~/NOV2021$ ls -l Final*
-rw-rw-r-- 1 afedorov afedorov 618734 мар 28 04:59 FinalAfrica_1
-rw-rw-r-- 1 afedorov afedorov 280705 мар 28 04:59 FinalAfrica_10
-rw-rw-r-- 1 afedorov afedorov 305577 мар 28 04:59 FinalAfrica_11
-rw-rw-r-- 1 afedorov afedorov 314769 мар 28 04:59 FinalAfrica_12
-rw-rw-r-- 1 afedorov afedorov 175959 мар 28 04:59 FinalAfrica_13
-rw-rw-r-- 1 afedorov afedorov 194772 мар 28 04:59 FinalAfrica_14
-rw-rw-r-- 1 afedorov afedorov 210933 мар 28 04:59 FinalAfrica_15
-rw-rw-r-- 1 afedorov afedorov 281346 мар 28 04:59 FinalAfrica_16
-rw-rw-r-- 1 afedorov afedorov 254607 мар 28 04:59 FinalAfrica_17
-rw-rw-r-- 1 afedorov afedorov 168675 мар 28 04:59 FinalAfrica_18
-rw-rw-r-- 1 afedorov afedorov 162444 мар 28 04:59 FinalAfrica_19
-rw-rw-r-- 1 afedorov afedorov 537349 мар 28 04:59 FinalAfrica_2
-rw-rw-r-- 1 afedorov afedorov 172259 мар 28 04:59 FinalAfrica_20
-rw-rw-r-- 1 afedorov afedorov 120987 мар 28 04:59 FinalAfrica_21
-rw-rw-r-- 1 afedorov afedorov 120806 мар 28 04:59 FinalAfrica_22
-rw-rw-r-- 1 afedorov afedorov 486291 мар 28 04:59 FinalAfrica_3
-rw-rw-r-- 1 afedorov afedorov 444500 мар 28 04:59 FinalAfrica_4
-rw-rw-r-- 1 afedorov afedorov 465854 мар 28 04:59 FinalAfrica_5
-rw-rw-r-- 1 afedorov afedorov 331425 мар 28 04:59 FinalAfrica_6
-rw-rw-r-- 1 afedorov afedorov 356565 мар 28 04:59 FinalAfrica_7
-rw-rw-r-- 1 afedorov afedorov 383661 мар 28 04:59 FinalAfrica_8
-rw-rw-r-- 1 afedorov afedorov 295573 мар 28 04:59 FinalAfrica_9
-rw-rw-r-- 1 afedorov afedorov  36855 мар 28 04:57 FinalAmerica_1
-rw-rw-r-- 1 afedorov afedorov  25332 мар 28 04:57 FinalAmerica_10
-rw-rw-r-- 1 afedorov afedorov  27948 мар 28 04:57 FinalAmerica_11
-rw-rw-r-- 1 afedorov afedorov  33204 мар 28 04:57 FinalAmerica_12
-rw-rw-r-- 1 afedorov afedorov   8401 мар 28 04:57 FinalAmerica_13
-rw-rw-r-- 1 afedorov afedorov  22686 мар 28 04:57 FinalAmerica_14
```

```
-rw-rw-r-- 1 afedorov afedorov  16685 мар 28 04:57 FinalAmerica_15
-rw-rw-r-- 1 afedorov afedorov  17109 мар 28 04:57 FinalAmerica_16
-rw-rw-r-- 1 afedorov afedorov  10189 мар 28 04:57 FinalAmerica_17
-rw-rw-r-- 1 afedorov afedorov  10505 мар 28 04:57 FinalAmerica_18
-rw-rw-r-- 1 afedorov afedorov   7742 мар 28 04:57 FinalAmerica_19
-rw-rw-r-- 1 afedorov afedorov  40863 мар 28 04:57 FinalAmerica_2
-rw-rw-r-- 1 afedorov afedorov   9631 мар 28 04:57 FinalAmerica_20
-rw-rw-r-- 1 afedorov afedorov   7370 мар 28 04:57 FinalAmerica_21
-rw-rw-r-- 1 afedorov afedorov   9881 мар 28 04:57 FinalAmerica_22
-rw-rw-r-- 1 afedorov afedorov  29183 мар 28 04:57 FinalAmerica_3
-rw-rw-r-- 1 afedorov afedorov  31140 мар 28 04:57 FinalAmerica_4
-rw-rw-r-- 1 afedorov afedorov  33882 мар 28 04:57 FinalAmerica_5
-rw-rw-r-- 1 afedorov afedorov  25600 мар 28 04:57 FinalAmerica_6
-rw-rw-r-- 1 afedorov afedorov  29343 мар 28 04:57 FinalAmerica_7
-rw-rw-r-- 1 afedorov afedorov  22123 мар 28 04:57 FinalAmerica_8
-rw-rw-r-- 1 afedorov afedorov  15800 мар 28 04:57 FinalAmerica_9
-rw-rw-r-- 1 afedorov afedorov   6425 мар 28 04:58 FinalEastAsia_1
-rw-rw-r-- 1 afedorov afedorov   6466 мар 28 04:58 FinalEastAsia_10
-rw-rw-r-- 1 afedorov afedorov  13188 мар 28 04:58 FinalEastAsia_11
-rw-rw-r-- 1 afedorov afedorov   6214 мар 28 04:58 FinalEastAsia_12
-rw-rw-r-- 1 afedorov afedorov   2395 мар 28 04:58 FinalEastAsia_13
-rw-rw-r-- 1 afedorov afedorov   5673 мар 28 04:58 FinalEastAsia_14
-rw-rw-r-- 1 afedorov afedorov  25633 мар 28 04:58 FinalEastAsia_15
-rw-rw-r-- 1 afedorov afedorov  15854 мар 28 04:58 FinalEastAsia_16
-rw-rw-r-- 1 afedorov afedorov   1960 мар 28 04:58 FinalEastAsia_17
-rw-rw-r-- 1 afedorov afedorov   2094 мар 28 04:58 FinalEastAsia_18
-rw-rw-r-- 1 afedorov afedorov   1286 мар 28 04:58 FinalEastAsia_19
-rw-rw-r-- 1 afedorov afedorov   8058 мар 28 04:58 FinalEastAsia_2
-rw-rw-r-- 1 afedorov afedorov    432 мар 28 04:58 FinalEastAsia_20
-rw-rw-r-- 1 afedorov afedorov    377 мар 28 04:58 FinalEastAsia_21
-rw-rw-r-- 1 afedorov afedorov    848 мар 28 04:58 FinalEastAsia_22
-rw-rw-r-- 1 afedorov afedorov  20405 мар 28 04:58 FinalEastAsia_3
-rw-rw-r-- 1 afedorov afedorov  11617 мар 28 04:58 FinalEastAsia_4
-rw-rw-r-- 1 afedorov afedorov   4678 мар 28 04:58 FinalEastAsia_5
-rw-rw-r-- 1 afedorov afedorov   3745 мар 28 04:58 FinalEastAsia_6
-rw-rw-r-- 1 afedorov afedorov   2299 мар 28 04:58 FinalEastAsia_7
-rw-rw-r-- 1 afedorov afedorov   4046 мар 28 04:58 FinalEastAsia_8
-rw-rw-r-- 1 afedorov afedorov   2029 мар 28 04:58 FinalEastAsia_9
-rw-rw-r-- 1 afedorov afedorov  60356 мар 28 04:58 FinalEurope_1
-rw-rw-r-- 1 afedorov afedorov 109950 мар 28 04:58 FinalEurope_10
-rw-rw-r-- 1 afedorov afedorov  68216 мар 28 04:58 FinalEurope_11
```

```
-rw-rw-r-- 1 afedorov afedorov  44735 мар 28 04:58 FinalEurope_12
-rw-rw-r-- 1 afedorov afedorov  58988 мар 28 04:58 FinalEurope_13
-rw-rw-r-- 1 afedorov afedorov  43285 мар 28 04:58 FinalEurope_14
-rw-rw-r-- 1 afedorov afedorov  47169 мар 28 04:58 FinalEurope_15
-rw-rw-r-- 1 afedorov afedorov  22366 мар 28 04:58 FinalEurope_16
-rw-rw-r-- 1 afedorov afedorov  46130 мар 28 04:58 FinalEurope_17
-rw-rw-r-- 1 afedorov afedorov  54384 мар 28 04:58 FinalEurope_18
-rw-rw-r-- 1 afedorov afedorov   9280 мар 28 04:58 FinalEurope_19
-rw-rw-r-- 1 afedorov afedorov  68573 мар 28 04:58 FinalEurope_2
-rw-rw-r-- 1 afedorov afedorov  32177 мар 28 04:58 FinalEurope_20
-rw-rw-r-- 1 afedorov afedorov   9570 мар 28 04:58 FinalEurope_21
-rw-rw-r-- 1 afedorov afedorov  16612 мар 28 04:58 FinalEurope_22
-rw-rw-r-- 1 afedorov afedorov  76950 мар 28 04:58 FinalEurope_3
-rw-rw-r-- 1 afedorov afedorov  58887 мар 28 04:58 FinalEurope_4
-rw-rw-r-- 1 afedorov afedorov  51787 мар 28 04:58 FinalEurope_5
-rw-rw-r-- 1 afedorov afedorov  64694 мар 28 04:58 FinalEurope_6
-rw-rw-r-- 1 afedorov afedorov  37134 мар 28 04:58 FinalEurope_7
-rw-rw-r-- 1 afedorov afedorov  56939 мар 28 04:58 FinalEurope_8
-rw-rw-r-- 1 afedorov afedorov  48349 мар 28 04:58 FinalEurope_9
-rw-rw-r-- 1 afedorov afedorov  35976 мар 28 04:57 FinalOceania_1
-rw-rw-r-- 1 afedorov afedorov    552 мар 28 04:57 FinalOceania_10
-rw-rw-r-- 1 afedorov afedorov    262 мар 28 04:57 FinalOceania_11
-rw-rw-r-- 1 afedorov afedorov  15325 мар 28 04:57 FinalOceania_12
-rw-rw-r-- 1 afedorov afedorov    890 мар 28 04:57 FinalOceania_13
-rw-rw-r-- 1 afedorov afedorov   2977 мар 28 04:57 FinalOceania_14
-rw-rw-r-- 1 afedorov afedorov   7703 мар 28 04:57 FinalOceania_15
-rw-rw-r-- 1 afedorov afedorov  16842 мар 28 04:57 FinalOceania_16
-rw-rw-r-- 1 afedorov afedorov   6375 мар 28 04:57 FinalOceania_17
-rw-rw-r-- 1 afedorov afedorov   7439 мар 28 04:57 FinalOceania_18
-rw-rw-r-- 1 afedorov afedorov  13609 мар 28 04:57 FinalOceania_19
-rw-rw-r-- 1 afedorov afedorov   1726 мар 28 04:57 FinalOceania_2
-rw-rw-r-- 1 afedorov afedorov   1782 мар 28 04:57 FinalOceania_20
-rw-rw-r-- 1 afedorov afedorov   1391 мар 28 04:57 FinalOceania_21
-rw-rw-r-- 1 afedorov afedorov    384 мар 28 04:57 FinalOceania_22
-rw-rw-r-- 1 afedorov afedorov  44722 мар 28 04:57 FinalOceania_3
-rw-rw-r-- 1 afedorov afedorov  10243 мар 28 04:57 FinalOceania_4
-rw-rw-r-- 1 afedorov afedorov   5241 мар 28 04:57 FinalOceania_5
-rw-rw-r-- 1 afedorov afedorov  17898 мар 28 04:57 FinalOceania_6
-rw-rw-r-- 1 afedorov afedorov    426 мар 28 04:57 FinalOceania_7
-rw-rw-r-- 1 afedorov afedorov   4457 мар 28 04:57 FinalOceania_8
-rw-rw-r-- 1 afedorov afedorov    522 мар 28 04:57 FinalOceania_9
```
