# Supplementary material for: Analysis of Common SNPs across Continents Reveals Major Genomic Differences between Human Populations
Source: Genes (Basel). 2022 Aug 18;13(8):1472. doi: 10.3390/genes13081472 (PMC9408407; doi:10.3390/genes13081472)
Supplement: Supplementary file 1 [file genes-13-01472-s001.zip › genes-1766591-supplementary/ATLAS3_notebook_protocols.pdf]

ATLAS3 PROTOCOLS

September 11, 2020

```
grep -e Kho_AFR  -e Con_AFR -e JuX_AFR Cha_XXX_Chagyrskaya_dat3
 1563  grep  Pap_OCE  Cha_XXX_Chagyrskaya_dat3
 1564  grep FIN_EUR  Cha_XXX_Chagyrskaya_dat3
 1565  grep -e Kho_AFR  -e Con_AFR -e JuX_AFR Cha_XXX_Chagyrskaya_dat3
>ATLAS3_AFR_Cha_XXX
 1566  grep  Pap_OCE  Cha_XXX_Chagyrskaya_dat3 >ATLAS3_OCE_Cha_XXX
 1567  grep FIN_EUR  Cha_XXX_Chagyrskaya_dat3 >ATLAS3_FIN_Cha_XXX
 1568  pwd
 1569  mv ATLAS3* ..
 1570  exit
 1571  cd MAY2020/
 1572  ls
 1573  cd DATAjune2020
 1574  history
 1575  grep -e TSI_EUR -e IBS_EUR  Cha_XXX_Chagyrskaya_dat3
 1576  grep -e TSI_EUR -e IBS_EUR  Cha_XXX_Chagyrskaya_dat3 >A3_TSI_IBS_Cha_XXX
 1577  ls Ust_XXX*
 1578  grep -e TSI_EUR -e IBS_EUR  Ust_XXX_Ustishim_dat3 >A3_TSI_IBS_Ust_XXX
 1579  history
afedorov@bio:~/MAY2020/DATAjune2020$  grep -e Kho_AFR  -e Con_AFR -e JuX_AFR
Ust_XXX_Ustishim_dat3 >A3_AFR_Ust_XXX
afedorov@bio:~/MAY2020/DATAjune2020$  grep  Pap_OCE  Ust_XXX_Ustishim_dat3
>A3_OCE_Ust_XXX
afedorov@bio:~/MAY2020/DATAjune2020$ grep -e FIN_EUR -e CEU_EUR
Ust_XXX_Ustishim_dat3 >A3_FIN_CEU_Ust_XX
afedorov@bio:~/MAY2020/DATAjune2020$ ls -l A3_*
-rw-rw-r-- 1 afedorov afedorov   4445 сен 12 02:23 A3_AFR_Ust_XXX
-rw-rw-r-- 1 afedorov afedorov   4456 сен 12 02:25 A3_FIN_CEU_Ust_XX
-rw-rw-r-- 1 afedorov afedorov   4693 сен 12 02:24 A3_OCE_Ust_XXX
-rw-rw-r-- 1 afedorov afedorov 145056 сен 12 02:21 A3_TSI_IBS_Cha_XXX
-rw-rw-r-- 1 afedorov afedorov   9372 сен 12 02:22 A3_TSI_IBS_Ust_XXX
afedorov@bio:~/MAY2020/DATAjune2020$ mv A3_* ..
```

November 20, 2020

Mapping Neanderthal's IBDs on Chromosome-1

```
/home/afedorov/AI3
afedorov@bio:~/AI3$ ls
NeanderthalIBDdistribution.pl  TESTING  TRAINING
#!/usr/local/perl
%H=(); #hash of starting position IBD => length IBD
@N = (); #array starting positions of IBDs
$c=$c2=0; #counter

open(IN, "/home/afedorov/MAY2020/DATAjune2020/Cha_XXX_Chagyrskaya_dat3");
while(<IN>) {
	chop;
	@a=split(/\t/, $_);
	if ($a[0] =~/CHR1\b/) {
		#	print "$a[1]\t$a[8]\n";
		unless($H{$a[1]}) {$H{$a[1]}=$a[8]; push(@N,$a[1]);}
		else {$H{$a[1]} .="\t" . $a[8];}

	}
}
@S = sort { $a <=> $b } @N;
$END = 0;
for $x (0..$#S) {
	@b=split("\t", $H{$S[$x]});
	@s = sort { $a <=> $b } @b;
	$max =$s[$#s];
	$end = $S[$x] + $max;
	$c++;
	if ($end <=$END) {next;}
	else {
		$END =$end;
		print $S[$x], "\t", $max, "\n";
		$c2++;
	}
}
print "Number of rows is $c new C2 is $c2\n";
```

Studying IBDs between AFR and OCE or EAS:

2069 cd MAY2020/
2070 ls
2071 cd DATAjune2020
2072 ls
2073 grep EAS MSL_AFR_HG03095_dat3
2074 grep EAS MSL_AFR_*_dat3
2075 grep EAS MSL_AFR_*_dat3 |grep CHR3
2076 grep OCE MSL_AFR_*_dat3 |grep CHR3
2077 history

November 25, 2020

American-specific SNPs

Studied 8 people from 4 American tribes:

grep Kar simon_pops2_A2.txt	3 inds
grep Cha simon_pops2_A2.txt	1 ind
grep Sur simon_pops2_A2.txt	2 inds
grep Pia simon_pops2_A2.txt	2 inds

```
2033  vi  AmericanSNPs2020.pl
 2034  perl  AmericanSNPs2020.pl 1
 2035  history
afedorov@bio:~/MAY2020$ pwd
/home/afedorov/MAY2020
afedorov@bio:~/MAY2020$ ls -l AMR*
-rw-rw-r-- 1 afedorov afedorov 36513 ноя 26 05:51 AMR_SNPs_1
afedorov@bio:~/MAY2020$ wc  AMR*
 1111  6666 36513 AMR_SNPs_1
```

CONCLUSION: on Chr 1 I found 1111 SNPs that occurred >=3 times in 8 americans and nobody else from Simon project

Changed program. Now the requirements are the following:

SNPs that occurred >=3 times in 8 americans Cha,Kar,Pia,Sur and nobody else outside AMERICA from Simon project. The modified program allows matching also in American populations: May, Mix, Pim, Que, Zap.

```
afedorov@bio:~/MAY2020$ wc  AMR*
```

2425 16975 84074 AMR_SNPs_1

Final change: I also allowed matching SNPs to ARC populations. The results are the following:
afedorov@bio:~/MAY2020$ wc AMR*
3502 31518 136353

afedorov@bio:~/MAY2020$ more *AMR_SNPs_1*

6	3	0	9	CHR1	51802	s1x51802	C	T
6	2	0	8	CHR1	84139	rs183605470	A	T
3	0	0	3	CHR1	239974	s1x239974	C	T
4	0	0	4	CHR1	461955	s1x461955	G	A
4	6	0	10	CHR1	547914	s1x547914	G	T
4	4	0	8	CHR1	548224	s1x548224	C	A
5	1	1	7	CHR1	551088	s1x551088	G	T
4	0	0	4	CHR1	662577	rs369208112	A	G
8	5	0	13	CHR1	826205	rs150036925	G	A
3	4	1	8	CHR1	891049	rs117770195	C	T
8	6	0	14	CHR1	908275	rs145574509	G	A
8	6	0	14	CHR1	920890	rs181925002	A	G
3	5	1	9	CHR1	1094888	rs182013341	C	T
12	11	2	25	CHR1	1124792	rs143677262	A	T
11	12	2	25	CHR1	1163767	rs182022102	C	T
12	11	2	25	CHR1	1201632	rs12402622	G	A

Where Col1 = S.Amer tribes; Col2 = Mexicans; Col3 = ARC (#alleles)

November 26, 2020

Now my goal is to take *AMR_SNPs_1* data and check the presence of these alleles in 1000G datasets.

AmericaSpecificSNPs file:

rs186084397	4<br>0	8<br>0	0	12	CHR1	5995845	rs186084397	G	A	77	1	1
rs146736582	4<br>0	17<br>0	0	21	CHR1	6243367	rs146736582	G	A	102	0	0
rs999262	3<br>0	4<br>0	1	8	CHR1	6360962	rs999262	T	A	24	0	0
rs139864117	4<br>0	7<br>0	2	13	CHR1	6574244	rs139864117	C	T	65	0	0
rs138726316	4<br>0	7<br>0	2	13	CHR1	6582065	rs138726316	A	C	68	0	0
rs147976585	4<br>0	7<br>0	2	13	CHR1	6587762	rs147976585	G	A	68	0	0
rs189128312	4<br>0	7<br>0	2	13	CHR1	6598567	rs189128312	A	T	69	0	0
rs559091938	4<br>0	0<br>0	0	4	CHR1	6870255	s1x6870255	T	A	18	1	0
rs184440184	4<br>0	0<br>0	0	4	CHR1	6949528	rs184440184	G	A	18	1	1

IN this output file (AmericaSpecificSNPs) the last 5 columns are

1) **Number of $amr**
2) **Number of Eur**
3) **Number of Asia**
4) **Number of Afr**
5) **Number of Ind**

All in all, I got 1558 American-specific SNPs on Chr1 that have rs-IDs and are present in 1000G

number of nonfound SNPs in 1000G is 979 (those that present in Simons and absent in 1000G)

The present pipeline:

1) Step 1:   "perl AmericanSNPs2020.pl 1" this command produces the *AMR_SNPs_1* output file for chromosome 1
2) Step 2:   "perl AmericanSNPs1000G.pl 1" this program produces the *AmericaSpecificSNPs* output file for chromosome 1.

Finally, I need to look at these American-specific alleles in Estonian project (Maybe they are enriched in Arctic, Siberia, or other populations that may point to the origins of Americans.

November 27, 2020

afedorov@bio:~/MAY2020$ perl **AmericanSNPsESTONIA.pl**   1

410   number of items is 402
395   Cac_AMR
336   Wic_AMR
306   Col_AMR
59   Kor_ARC
57   Chu_ARC
51   Esk_ARC
11   EVk_SIB   13inds
11   Bur_SIB   17inds
8   Eve_SIB   8inds
5   Ket_SIB   3inds
4   For_SIB   3inds
3   Sho_SIB 2 inds
3   Cro_EUR Croats
3   Sel_SIB   3inds
3   Kyr_CAS 7
3   Sak_SIB 7inds
2   Alt_CAS 6
2   BUm_SAS Burmise Maynamar
2   Udm_EUR 4
2   BSh_EUR   5
2   Vie_EAS 10
2   Kha_SIB 3inds

2	Tuv_SIB 3inds
2	Kaz_CAS 3
1	Kab_CAU
1	Rus_CAS
1	Agt_OCE
1	Viz_OCE
1	Tat_EUR
1	Mar_EUR
1	Kry_EUR
1	Abk_CAU
1	Baj_OCE
1	Mis_EUR
1	Ish_CAS

OUTPUT Datafile: **AmericaSpecificSNPsEstonia_1**

```
rs186485779	5	4	1	10	CHR1	1605033	rs186485779	C	T	57	0	0
	0	0		Wic_AMR	Kor_ARC	Kor_ARC	Kor_ARC

rs192491441	4	1	0	5	CHR1	2046229	rs192491441	A	G	46	1	0
	0	0		Col_AMR
```

Starting pipeline for calculation American-specific SNPs:

perl AmericanSNPstart1.pl (running AmericanSNPs2020.pl on 22 Chr)

perl AmericanSNPstart2.pl (running AmericanSNPs1000G.pl on 22 Chr)

RESULTS:

```
afedorov@bio:~/MAY2020$ wc AmericaSpecificSNPs_1
  1610  88850 359157 AmericaSpecificSNPs_1
afedorov@bio:~/MAY2020$ wc AmericaSpecificSNPs_2
  1915 103754 419697 AmericaSpecificSNPs_2
afedorov@bio:~/MAY2020$ wc AmericaSpecificSNPs_3
  1354  70857 286362 AmericaSpecificSNPs_3
afedorov@bio:~/MAY2020$ wc AmericaSpecificSNPs_4
  1379  74348 300386 AmericaSpecificSNPs_4
afedorov@bio:~/MAY2020$ wc AmericaSpecificSNPs_5
  1473  78992 319169 AmericaSpecificSNPs_5
afedorov@bio:~/MAY2020$ wc AmericaSpecificSNPs_6
  1198  64048 258707 AmericaSpecificSNPs_6
afedorov@bio:~/MAY2020$ wc AmericaSpecificSNPs_7
  1224  68509 276558 AmericaSpecificSNPs_7
afedorov@bio:~/MAY2020$ wc AmericaSpecificSNPs_8
  1287  65909 266050 AmericaSpecificSNPs_8
afedorov@bio:~/MAY2020$ wc AmericaSpecificSNPs_9
   944  47857 193357 AmericaSpecificSNPs_9
afedorov@bio:~/MAY2020$ wc AmericaSpecificSNPs_10
```

```
  1040  57132 231628 AmericaSpecificSNPs_10
afedorov@bio:~/MAY2020$ wc AmericaSpecificSNPs_11
  1002  57524 233132 AmericaSpecificSNPs_11
afedorov@bio:~/MAY2020$ wc AmericaSpecificSNPs_12
  1018  59249 240071 AmericaSpecificSNPs_12
afedorov@bio:~/MAY2020$ wc AmericaSpecificSNPs_13
   697  35887 145622 AmericaSpecificSNPs_13
afedorov@bio:~/MAY2020$ wc AmericaSpecificSNPs_14
   750  46215 187111 AmericaSpecificSNPs_14
afedorov@bio:~/MAY2020$ wc AmericaSpecificSNPs_15
   655  35858 145374 AmericaSpecificSNPs_15
afedorov@bio:~/MAY2020$ wc AmericaSpecificSNPs_16
   691  38029 153979 AmericaSpecificSNPs_16
afedorov@bio:~/MAY2020$ wc AmericaSpecificSNPs_17
   575  30815 124792 AmericaSpecificSNPs_17
afedorov@bio:~/MAY2020$ wc AmericaSpecificSNPs_18
   604  31308 126776 AmericaSpecificSNPs_18
afedorov@bio:~/MAY2020$ wc AmericaSpecificSNPs_19
  375 19712 79872 AmericaSpecificSNPs_19
afedorov@bio:~/MAY2020$ wc AmericaSpecificSNPs_20
  408 21751 88048 AmericaSpecificSNPs_20
afedorov@bio:~/MAY2020$ wc AmericaSpecificSNPs_21
  273 15228 61649 AmericaSpecificSNPs_21
afedorov@bio:~/MAY2020$ wc AmericaSpecificSNPs_22
  273 16454 66597 AmericaSpecificSNPs_22
```

All in all: I got 20,745 SNPs specific for Native Americans (files AmericaSpecificSNPs_1..22

Conclusion: We hypothesized that origin of Native americans may be far older than 25 Kya based on their 20K unique American-specific SNPs and very low Neanderthals. However, about 30% of Native Americans should be more recent admixture (~20Kya) with Arctic and Siberian populations.

Next goals:

1) Analyze heterozygosity index in Native Americans vs EUR, EAS, etc. from Simons
2) Analyze ancient Malta genome (hypothesized ancestor of Americans; Raghavan M. et al Nature 2014, 505(7481):87-91) and other ancient genomes related to relatives of Americans.

October 25, 2021

The present pipeline:

1) Step 1: "perl AmericanSNPs2020.pl 10" this command processes Simon VCF dataset file '/home/afedorov/simon/simon_chr'.$L.'.gz' and produces the *AMR_SNPs_10* output file for chromosome 10
2) Step 2: "perl AmericanSNPs1000G.pl 10" this program processes the *AMR_SNPs_10* file (created at the first step) and VCF for 1000G ('/home/afedorov/2500GENOMES/ALL.chr'.$L.'.phase3_shapeit2_mvncall_integrated_v5a.20130502.genotypes.vcf.gz') and produces the *AmericaSpecificSNPs_10* output file for chromosome 10. It counts the occurrences in $asia; $eur; $afr; $ind; and $amr populations
3) Step 3: "perl AmericanSNPEstonia.pl 10" this program processes the *AmericaSpecificSNPs_10* file and the Estonian VCF file () and produces the AmericaSpecificSNPsEstonia_10 output file for chromosome 10.

   It also produces two additional files: AmericaSpecificSNPsEstonia2_10 (most important) and AmericaSpecificSNPsEstonia3_10 shown below:

```
afedorov@bio:~/MAY2020$ more  AmericaSpecificSNPsEstonia3_22
53	Cac_AMR
49	Col_AMR
40	Wic_AMR
12	Kor_ARC
7	Chu_ARC
7	Ira_MDE
6	Esk_ARC
3	EVk_SIB
2	Ket_SIB
2	BSh_EUR
2	Tun_SIB
2	Udm_EUR
1	Eve_SIB
1	Alt_CAS
1	Kry_EUR
1	Kyr_CAS
1	Yag_CAS
1	Uyg_CAS
```

```
afedorov@bio:~/MAY2020$ more  AmericaSpecificSNPsEstonia2_22
```

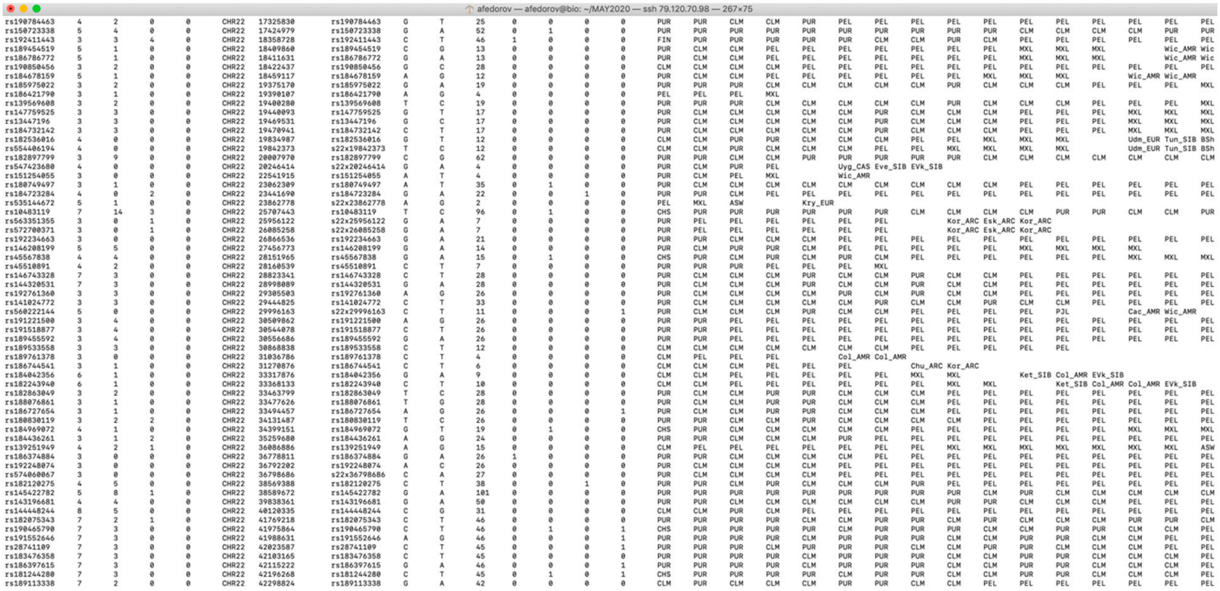

The file AmericaSpecificSNPsEstonia2_10 gives the complete information

HISTORY FOR TODAY:

```
cd MAY2020/
 2024  ls
 2025  ls -l *pl
 2026  ls -l AmericaSpecificSNPs*
 2027  less -S AmericaSpecificSNPs_1
 2028  ls -l *pl
 2029  vi AmericanSNPstart3.pl
 2030  vi AmericanSNPstart2.pl
 2031  vi AmericanSNPs2020.pl
 2032  less -S ids_simon
 2033  vi AmericanSNPs2020.pl
 2034  vi AmericanSNPsESTONIA.pl
 2035  less -S AmericaSpecificSNPsEstonia3_22
 2036  more  AmericaSpecificSNPsEstonia3_22
 2037  more  AmericaSpecificSNPsEstonia2_22
 2038  less -S  AmericaSpecificSNPsEstonia2_22
 2039  less -S  AmericaSpecificSNPsEstonia_22
 2040  less -S  AmericaSpecificSNPsEstonia2_22
 2041  ls -l *pl
 2042  vi AmericanSNPs2020.pl
 2043  iv AmericanSNPs1000G.pl
 2044  vi AmericanSNPs1000G.pl
 2045  vi AmericanSNPs2020.pl
 2046  ls
 2047  more simon_pops2_A2.txt
 2048  more simon_pops2_A2.txt |grep OCE
 2049  cp AmericanSNPs2020.pl OceaniaSimonStep1.pl
 2050  vi OceaniaSimonStep1.pl
 2051  more simon_pops2_A2.txt |grep Pap
 2052  more simon_pops2_A2.txt |grep Aus
 2053  more simon_pops2_A2.txt |grep Bou
 2054  more simon_pops2_A2.txt |grep Dus
 2055  more simon_pops2_A2.txt |grep Mao
 2056  more simon_pops2_A2.txt |grep Igo
 2057  more simon_pops2_A2.txt |grep Haw
 2058  vi OceaniaSimonStep1.pl
 2059  perl OceaniaSimonStep1.pl  10
 2060  ls
 2061  more OCE_SNPs_10
 2062  ls
```

```
 2063  more AMR_SNPs_10
 2064  vi OceaniaSimonStep1.pl
 2065  perl OceaniaSimonStep1.pl  10
 2066  more OCE_SNPs_10
 2067  history
```

October 28, 2021

Created a new program `Oceania1000gStep2new.pl`, which keeps SNPs missing in the 1000G.

```
2097  mkdir NOV2021
 2098  cd MAY2020/
 2099  ls -l *pl
 2100  ls Oce*pl
 2101  cp Oce*pl ../NOV2021/
 2102  ls
 2103  cp OCE_S* ../NOV2021/
 2104  ls Oce*pl
 2105  vi OceaniaSimonStep1.pl
 2106  cp simon_pops2_A2.txt ../NOV2021/
 2107  cp ids_simon ../NOV2021/
 2108  vi OceaniaSimonStep1.pl
 2109  vi Oceania1000gStep2.pl
 2110  cp 2504ids ../NOV2021/
 2111  vi Oceania1000gStep2.pl
 2112  cp igsr_samples.tsv ../NOV2021/
 2113  vi OceanEstoniaStep3.pl
 2114  cp estonian_pops2_A2.txt  ../NOV2021/
 2115  cd ../NOV2021/
 2116  ls
 2117  ls -l
 2118  cp Oceania1000gStep2.pl Oceania1000gStep2new.pl
 2119  vi Oceania1000gStep2new.pl
 2120  perl  Oceania1000gStep2new.pl 10
 2121  ls -l
 2122  wc OCE_SNPs_10
 2123  wc OceaniaSpecificSNPs_10
 2124  head OceaniaSpecificSNPs_10
 2125  vi Oceania1000gStep2new.pl
 2126  cd ../MAY2020/
 2127  ls
 2128  head  AMR_SNPs_7
 2129  ls -l *pl
 2130  vi AmericanSNPs1000G.pl
 2131  vi AmericanSNPs2020.pl
 2132  vi OceaniaSimonStep1.pl
 2133  head OCE_SNPs_10
 2134  head  AMR_SNPs_7
 2135  cd ../NOV2021/
 2136  ls
 2137  head OCE_SNPs_1
 2138  history
 2139  cd ../MAY2020/
 2140  ls
 2141  wc AMR_SNPs_10
 2142  wc AmericaSpecificSNPs_10
 2143  cp AmericaSpecificSNPs_10 AMR_SNPs_10 ../NOV2021/
 2144  ls -l *pl
 2145  cp AmericanSNPs1000G.pl ../NOV2021/
 2146  cd ../NOV2021/
 2147  ls
 2148  perl AmericanSNPs1000G.pl 10
 2149  ls
 2150  wc AmericaSpecificSNPs_10
 2151  wc AMR_SNPs_10
 2152  wc OceaniaSpecificSNPs_10
 2153  vi Oceania1000gStep2new.pl
```

```
2154  perl Oceania1000gStep2new.pl  10
2155  vi Oceania1000gStep2new.pl
2156  ls
2157  more OCE_SNPs_10
2158  vi Oceania1000gStep2new.pl
2159  perl Oceania1000gStep2new.pl  10
2160  vi Oceania1000gStep2new.pl
2161  perl Oceania1000gStep2new.pl  10
2162  vi Oceania1000gStep2new.pl
2163  perl Oceania1000gStep2new.pl  10
2164  vi Oceania1000gStep2new.pl
2165  perl Oceania1000gStep2new.pl  10
2166  vi Oceania1000gStep2new.pl
2167  perl Oceania1000gStep2new.pl  10
2168  vi Oceania1000gStep2new.pl
2169  perl Oceania1000gStep2new.pl  10
2170  vi Oceania1000gStep2new.pl
2171  perl Oceania1000gStep2new.pl  10
2172  vi Oceania1000gStep2new.pl
2173  perl Oceania1000gStep2new.pl  10
2174  vi Oceania1000gStep2new.pl
2175  perl Oceania1000gStep2new.pl  10
2176  ls
2177  moe OceaniaSpecificSNPs_10
2178  more OceaniaSpecificSNPs_10
2179  vi Oceania1000gStep2new.pl
2180  perl Oceania1000gStep2new.pl  10
2181  ls
2182  more OceaniaSpecificSNPs_10
2183  vi Oceania1000gStep2new.pl
2184  perl Oceania1000gStep2new.pl  10
2185  more OceaniaSpecificSNPs_10
2186  wc OceaniaSpecificSNPs_10
2187  more OceaniaSpecificSNPs_10
2188  ls
2189  ls -l *pl
2190  vi OceaniaSNPstart2.pl
2191  ls -l OCE_*
2192  perl OceaniaSNPstart2.pl
2193  history
```

```
#new addition Oct 28,2021
$r =0; # order of elements  in the @positions
$count_r =0;
while (<FILE>){
        $line_N++;
        if ($line_N > 253) {
                chomp($_);
                @e=split(/\t/, $_);
                #print "$e[1] \n";
                if ($positions[$r] ==$e[1]) {$r++;}
                while ($positions[$r] <$e[1]) {
                        if ($positions[$#positons]<$e[1]  || $r>= $#positions) {last;}
                        #       print "position $rows[$r] \n";
                        print OUT "$RSid[$r]\t$rows[$r]\t0\t0\t0\t0\t0\tNOT\n";
                        $r++; $count_r++;
                }
                #end of addition
```

October 29, 2021

Finishing calculation of Oceania-Specific SNPs in BIO: /home/afedorov/NOV2021

```
HISTORY:
vi  OceanEstoniaStep3.pl
 2214  top
 2215  ls -l *pl
 2216  perl OceanEstoniaStep3.pl 10
 2217  ls
```

```
 2218  more OceaniaSpecificSNPsEstonia2_10
 2219  wc OceaniaSpecificSNPsEstonia2_10
 2220  top
 2221  ls -l *pl
 2222  perl OceaniaSNPstart3.pl
```

```
fedorov@bio:~/NOV2021$ wc OceaniaSpecificSNPsEstonia2_1
  6033 110372 504191 OceaniaSpecificSNPsEstonia2_1
afedorov@bio:~/NOV2021$ wc OceaniaSpecificSNPsEstonia2_2
  4405  79961 365807 OceaniaSpecificSNPsEstonia2_2
afedorov@bio:~/NOV2021$ wc OceaniaSpecificSNPsEstonia2_3
  8315 152094 696776 OceaniaSpecificSNPsEstonia2_3
afedorov@bio:~/NOV2021$ wc OceaniaSpecificSNPsEstonia2_4
  3959  72393 329838 OceaniaSpecificSNPsEstonia2_4
afedorov@bio:~/NOV2021$ wc OceaniaSpecificSNPsEstonia2_5
  4870  88749 406097 OceaniaSpecificSNPsEstonia2_5
afedorov@bio:~/NOV2021$ wc OceaniaSpecificSNPsEstonia2_6
  5302  97234 446848 OceaniaSpecificSNPsEstonia2_6
afedorov@bio:~/NOV2021$ wc OceaniaSpecificSNPsEstonia2_7
  3189  57782 261710 OceaniaSpecificSNPsEstonia2_7
afedorov@bio:~/NOV2021$ wc OceaniaSpecificSNPsEstonia2_8
  3781  69456 316880 OceaniaSpecificSNPsEstonia2_8
afedorov@bio:~/NOV2021$ wc OceaniaSpecificSNPsEstonia2_9
  2422  43756 198045 OceaniaSpecificSNPsEstonia2_9
afedorov@bio:~/NOV2021$ wc OceaniaSpecificSNPsEstonia2_10
  2289  41743 193560 OceaniaSpecificSNPsEstonia2_10
afedorov@bio:~/NOV2021$ wc OceaniaSpecificSNPsEstonia2_11
  2247  40905 190310 OceaniaSpecificSNPsEstonia2_11
afedorov@bio:~/NOV2021$ wc OceaniaSpecificSNPsEstonia2_12
  3707  68123 317727 OceaniaSpecificSNPsEstonia2_12
afedorov@bio:~/NOV2021$ wc OceaniaSpecificSNPsEstonia2_13
  2340  42413 196920 OceaniaSpecificSNPsEstonia2_13
afedorov@bio:~/NOV2021$ wc OceaniaSpecificSNPsEstonia2_14
  3295  59418 272788 OceaniaSpecificSNPsEstonia2_14
afedorov@bio:~/NOV2021$ wc OceaniaSpecificSNPsEstonia2_15
  2454  44726 206853 OceaniaSpecificSNPsEstonia2_15
afedorov@bio:~/NOV2021$ wc OceaniaSpecificSNPsEstonia2_16
  3253  59354 275244 OceaniaSpecificSNPsEstonia2_16
afedorov@bio:~/NOV2021$ wc OceaniaSpecificSNPsEstonia2_17
  2473  45639 212181 OceaniaSpecificSNPsEstonia2_17
afedorov@bio:~/NOV2021$ wc OceaniaSpecificSNPsEstonia2_18
  2387  43446 198409 OceaniaSpecificSNPsEstonia2_18
afedorov@bio:~/NOV2021$ wc OceaniaSpecificSNPsEstonia2_19
  1981  36241 166428 OceaniaSpecificSNPsEstonia2_19
afedorov@bio:~/NOV2021$ wc OceaniaSpecificSNPsEstonia2_20
  2354  42945 197809 OceaniaSpecificSNPsEstonia2_20
afedorov@bio:~/NOV2021$ wc OceaniaSpecificSNPsEstonia2_21
  1930  36043 169659 OceaniaSpecificSNPsEstonia2_21
afedorov@bio:~/NOV2021$ wc OceaniaSpecificSNPsEstonia2_22
  1448  26928 126393 OceaniaSpecificSNPsEstonia2_22
```

Sum on all chromosomes: **74,434** Oceania Specific SNPs
Which frequency is > 13% in Oceania (5 or more counts among 19 PapuaNewGuinea people) and <0.2% in the rest of the world.
For 25% threshold in Oceania, the counts will be <20,000.
COMMENTS:

1) For America-Specific SNPs I used threshold of >18% (3 counts among 8 people), which is higher than for Oceania. Secondly for AmericaSpecific I removed all SNPs missing in 1000G. Some SNPs may be inside areas, not sequenced in 1000G, so we have some underestimations for America.

Now I need to calculate China-Specific SNPs. Here are populations from Simon's project:

```
EAS   Cam   LP6005441-DNA_H03
EAS   Cam   LP6005441-DNA_G03
EAS   Han   SS6004469
EAS   Dai   SS6004467
EAS   Dai   LP6005592-DNA_D03
EAS   TuX   LP6005443-DNA_H01
EAS   She   LP6005443-DNA_G01
EAS   She   LP6005443-DNA_F01
EAS   Nax   LP6005443-DNA_E09
EAS   Lah   LP6005443-DNA_E01
EAS   Xib   LP6005443-DNA_C02
EAS   Dai   LP6005443-DNA_B01
EAS   Tuj   LP6005443-DNA_A02
EAS   YiX   LP6005442-DNA_H01
EAS   YiX   LP6005442-DNA_G01
EAS   Xib   LP6005442-DNA_D01
EAS   Hez   LP6005441-DNA_H05
EAS   Hez   LP6005441-DNA_G05
EAS   Tuj   LP6005441-DNA_F12
EAS   Oro   LP6005441-DNA_F09
EAS   Mon   LP6005441-DNA_F08
EAS   Dau   LP6005441-DNA_F04
EAS   Oro   LP6005441-DNA_E09
EAS   Mon   LP6005441-DNA_E08
EAS   TuX   LP6005441-DNA_D12
EAS   Mia   LP6005441-DNA_D08
EAS   Han   LP6005441-DNA_D05
EAS   Dai   LP6005441-DNA_D04
EAS   Mia   LP6005441-DNA_C08
EAS   Han   LP6005441-DNA_C05
EAS   Nax   LP6005441-DNA_B09
EAS   Lah   LP6005441-DNA_B07
EAS   XXX   LP6005441-DNA_A09
EAS   Jap   LP6005592-DNA_C02
EAS   Jap   LP6005441-DNA_D06
EAS   Jap   LP6005441-DNA_C06
EAS   Kor   LP6005443-DNA_D06
EAS   Kor   LP6005443-DNA_C06
EAS   Bur   LP6005519-DNA_B06
EAS   Bur   LP6005519-DNA_A06
EAS   Ami   LP6005443-DNA_G05
EAS   Ata   LP6005442-DNA_E07
EAS   Ami   LP6005442-DNA_C07
EAS   Tha   LP6005443-DNA_B07
EAS   Tha   LP6005443-DNA_A07
EAS   Kin   LP6005442-DNA_D11
EAS   Kin   LP6005442-DNA_C11
```

I will use 22 individuals with > 84% of China (Table 4, Atlas2)

```
afedorov@bio:~/NOV2021$ grep Dai simon_pops2_A2.txt
EAS   Dai   SS6004467
EAS   Dai   LP6005592-DNA_D03
EAS   Dai   LP6005443-DNA_B01
EAS   Dai   LP6005441-DNA_D04
afedorov@bio:~/NOV2021$ grep Han simon_pops2_A2.txt
EAS   Han   SS6004469
EAS   Han   LP6005441-DNA_D05
EAS   Han   LP6005441-DNA_C05
afedorov@bio:~/NOV2021$ grep Jap simon_pops2_A2.txt
EAS   Jap   LP6005592-DNA_C02
EAS   Jap   LP6005441-DNA_D06
EAS   Jap   LP6005441-DNA_C06
afedorov@bio:~/NOV2021$ grep Kor simon_pops2_A2.txt
EAS   Kor   LP6005443-DNA_D06
EAS   Kor   LP6005443-DNA_C06
afedorov@bio:~/NOV2021$ grep Mia simon_pops2_A2.txt
EAS   Mia   LP6005441-DNA_D08
EAS   Mia   LP6005441-DNA_C08
afedorov@bio:~/NOV2021$ grep Nax simon_pops2_A2.txt
EAS   Nax   LP6005443-DNA_E09
```

```
EAS   Nax   LP6005441-DNA_B09
afedorov@bio:~/NOV2021$ grep She simon_pops2_A2.txt
EAS   She   LP6005443-DNA_G01
EAS   She   LP6005443-DNA_F01
afedorov@bio:~/NOV2021$ grep Tuj simon_pops2_A2.txt
EAS   Tuj   LP6005443-DNA_A02
EAS   Tuj   LP6005441-DNA_F12
afedorov@bio:~/NOV2021$ grep YiX simon_pops2_A2.txt
EAS   YiX   LP6005442-DNA_H01
EAS  YiX       LP6005442-DNA_G01
```

The programs are the following:

```
afedorov@bio:~/NOV2021$ cp AmericanSNPs2020.pl ChinaSimonStep1.pl
afedorov@bio:~/NOV2021$ cp AmericanSNPs1000G.pl China1000gStep2.pl
afedorov@bio:~/NOV2021$ cp AmericanSNPsESTONIA.pl ChinaEstoniaStep3.pl
```

I finished calculation of China-Specific SNPs and got surprising results: There are considerably less such CHI-specific SNPs than OCE and AMR-specific ones. Below are possible reasons and explanations.

1) At the last (third) step the output of ChinaEstoniaStep3.pl gave <100 CHI-Specific SNPs, while AMR- has 20,000 and OCE- has 74,000. The problem may be that Estonian Database have only a few people from EAS (4 Vietnamese and 2 other EAS individuals). Maybe these EAS has been poorly sequenced, and many China-specific SNPs are missing in Estonian Database. Maybe Vietnamese do not have much CHI-specific (I doubt, because in Atlas2 Vietnamese are 78% CHI). Maybe there is a bug in spelling in the Step1 program. ANYWAY, we should not consider ChinaSpecificSNPsEstonia2_$L files, because they are nearly empty. Instead we should use the output from the previous step: files ChinaSpecificSNPs_$L files, which has in total **2,885** SNPs.
2) To clarify the puzzle with very low China-Specific SNPs, I need to analyze only 1000G Dataset and compare CHI-specific vs AMR-specific. Because EAS and AMR are well represented there and the rest populations from 1000G are pretty far from these two groups (no SIB or ARC, etc.). I need to write a special program to do this test based on the AmericanSNPs1000G.pl program.
3) Anyway, the results are very strange, taking into account that CHI-group are not related to EUR, MDE, AFR, AMR. It could be a clue to population puzzles.

```
afedorov@bio:~/NOV2021$ wc ChinaSpecificSNPs_1
  180 23814 95553 ChinaSpecificSNPs_1
afedorov@bio:~/NOV2021$ wc ChinaSpecificSNPs_2
   245  32259 129522 ChinaSpecificSNPs_2
afedorov@bio:~/NOV2021$ wc ChinaSpecificSNPs_3
   228  34922 140157 ChinaSpecificSNPs_3
afedorov@bio:~/NOV2021$ wc ChinaSpecificSNPs_4
   242  35704 143332 ChinaSpecificSNPs_4
afedorov@bio:~/NOV2021$ wc ChinaSpecificSNPs_5
   234  31632 127011 ChinaSpecificSNPs_5
afedorov@bio:~/NOV2021$ wc ChinaSpecificSNPs_6
  130 16270 65289 ChinaSpecificSNPs_6
afedorov@bio:~/NOV2021$ wc ChinaSpecificSNPs_7
   209  27194 109206 ChinaSpecificSNPs_7
afedorov@bio:~/NOV2021$ wc ChinaSpecificSNPs_8
  118 15898 63752 ChinaSpecificSNPs_8
afedorov@bio:~/NOV2021$ wc ChinaSpecificSNPs_9
  112 13571 54483 ChinaSpecificSNPs_9
afedorov@bio:~/NOV2021$ wc ChinaSpecificSNPs_10
  118 14923 59999 ChinaSpecificSNPs_10
afedorov@bio:~/NOV2021$ wc ChinaSpecificSNPs_11
  136 20551 82544 ChinaSpecificSNPs_11
afedorov@bio:~/NOV2021$ wc ChinaSpecificSNPs_12
  137 19052 76558 ChinaSpecificSNPs_12
```

```
afedorov@bio:~/NOV2021$ wc ChinaSpecificSNPs_13
   82 11209 44998 ChinaSpecificSNPs_13
afedorov@bio:~/NOV2021$ wc ChinaSpecificSNPs_14
  122 16520 66392 ChinaSpecificSNPs_14
afedorov@bio:~/NOV2021$ wc ChinaSpecificSNPs_15
   154  28353 113896 ChinaSpecificSNPs_15
afedorov@bio:~/NOV2021$ wc ChinaSpecificSNPs_16
  135 21764 87423 ChinaSpecificSNPs_16
afedorov@bio:~/NOV2021$ wc ChinaSpecificSNPs_17
   58  7537 30276 ChinaSpecificSNPs_17
afedorov@bio:~/NOV2021$ wc ChinaSpecificSNPs_18
   64  8532 34264 ChinaSpecificSNPs_18
afedorov@bio:~/NOV2021$ wc ChinaSpecificSNPs_19
   55  7295 29298 ChinaSpecificSNPs_19
afedorov@bio:~/NOV2021$ wc ChinaSpecificSNPs_20
   67  8482 34055 ChinaSpecificSNPs_20
afedorov@bio:~/NOV2021$ wc ChinaSpecificSNPs_21
  19 2444 9818 ChinaSpecificSNPs_21
afedorov@bio:~/NOV2021$ wc ChinaSpecificSNPs_22
   40  5468 21975 ChinaSpecificSNPs_22
```

October 31, 2021

Starting AFR-Specific SNP calculations.

```
afedorov@bio:~/NOV2021$ cp AmericanSNPs2020.pl AfricaSimonStep1.pl
afedorov@bio:~/NOV2021$ cp AmericanSNPs1000G.pl Africa1000gStep2.pl
afedorov@bio:~/NOV2021$ cp AmericanSNPsESTONIA.pl AfricaEstoniaStep3.pl
```

checking AFR populations

```
afedorov@bio:~/NOV2021$ grep   Gam simon_pops2_A2.txt
AFR   Gam   LP6005442-DNA_H10
AFR   Gam   LP6005442-DNA_G10
afedorov@bio:~/NOV2021$ grep   Luo simon_pops2_A2.txt
AFR   Luo   LP6005677-DNA_G01
AFR   Luo   LP6005442-DNA_F09
afedorov@bio:~/NOV2021$ grep Mas simon_pops2_A2.txt
AFR   Mas   LP6005443-DNA_F06
AFR   Mas   LP6005443-DNA_E06
afedorov@bio:~/NOV2021$ grep Luh simon_pops2_A2.txt
AFR   Luh   LP6005442-DNA_F11
AFR   Luh   LP6005442-DNA_E11
afedorov@bio:~/NOV2021$ grep Som simon_pops2_A2.txt
AFR   Som   LP6005442-DNA_D09
afedorov@bio:~/NOV2021$ grep JuX simon_pops2_A2.txt
AFR   JuX   SS6004473
AFR   JuX   LP6005443-DNA_G08
AFR   JuX   LP6005441-DNA_B11
AFR   JuX   LP6005441-DNA_A11
afedorov@bio:~/NOV2021$ grep Yor simon_pops2_A2.txt
AFR   Yor   SS6004475
AFR   Yor   LP6005442-DNA_B02
AFR   Yor   LP6005442-DNA_A02
afedorov@bio:~/NOV2021$ grep Esa simon_pops2_A2.txt
AFR   Esa   LP6005442-DNA_B10
AFR   Esa   LP6005442-DNA_A10
afedorov@bio:~/NOV2021$ grep Man simon_pops2_A2.txt
SIB   Man   LP6005443-DNA_G04
SIB   Man   LP6005443-DNA_F04
AFR   Man   SS6004470
AFR   Man   LP6005441-DNA_F07
AFR   Man   LP6005441-DNA_E07
afedorov@bio:~/NOV2021$ grep Men simon_pops2_A2.txt
AFR   Men   LP6005442-DNA_H11
AFR   Men   LP6005442-DNA_G11
afedorov@bio:~/NOV2021$ grep Kho  simon_pops2_A2.txt
SAS   Kho   LP6005519-DNA_E05
AFR   Kho   LP6005677-DNA_D03
```

```
AFR   Kho   LP6005592-DNA_C05
afedorov@bio:~/NOV2021$ grep Din  simon_pops2_A2.txt
AFR   Din   SS6004480
AFR   Din   LP6005443-DNA_H08
AFR   Din   LP6005443-DNA_B09
afedorov@bio:~/NOV2021$ grep Sah  simon_pops2_A2.txt
AFR   Sah   LP6005619-DNA_C01
AFR   Sah   LP6005619-DNA_B01
afedorov@bio:~/NOV2021$ grep Mbu  simon_pops2_A2.txt
AFR   Mbu   SS6004471
AFR   Mbu   LP6005592-DNA_C03
AFR   Mbu   LP6005441-DNA_B08
AFR   Mbu   LP6005441-DNA_A08
afedorov@bio:~/NOV2021$ grep Bia  simon_pops2_A2.txt
AFR   Bia   LP6005441-DNA_H02
AFR   Bia   LP6005441-DNA_G02
afedorov@bio:~/NOV2021$ grep Ban  simon_pops2_A2.txt
AFR   Ban   LP6005443-DNA_G02
AFR   Ban   LP6005443-DNA_F02
AFR   Ban   LP6005443-DNA_E02
AFR   Ban   LP6005441-DNA_F01
AFR   Ban   LP6005443-DNA_A01
AFR   Ban   LP6005441-DNA_B02
afedorov@bio:~/NOV2021$ grep Moz  simon_pops2_A2.txt
AFR   Moz   LP6005441-DNA_H08
AFR   Moz   LP6005441-DNA_G08
```

There are duplicates from other continents in Kho and Man

HISTORY:

```
2164  grep Cam  simon_pops2_A2.txt history
 2165  history
 2166  vi ChinaSimonStep1.pl
 2167  vi AfricaSimonStep1.pl
 2168  perl AfricaSimonStep1.pl 10
 2169  ls
 2170  wc AFR_SNPs_10
 2171  vi AfricaSimonStep1.pl
 2172  perl AfricaSimonStep1.pl 10
 2173  wc AFR_SNPs_10
 2174  ls -l *pl
 2175  cp  AmericanSNPstart1.pl
 2176  cp  AmericanSNPstart1.pl  AfricanSNPstart1.pl
 2177  vi AfricanSNPstart1.pl
 2178  perl AfricanSNPstart1.pl
 2179  top
 2180  ls
 2181  vi Africa1000gStep2.pl
 2182  perl Africa1000gStep2.pl  10
 2183  ls
 2184  wc AfricanSpecificSNPs_10
 2185  ls -l Afr*
 2186  vi Africa1000gStep2.pl
 2187  perl Africa1000gStep2.pl  10
 2188  ls
 2189  wc AfricanSpecificSNPs_10
 2190  ls -S AfricanSpecificSNPs_10
 2191  less -S AfricanSpecificSNPs_10
 2192  vi Africa1000gStep2.pl
 2193  perl Africa1000gStep2.pl  10
 2194  wc AfricanSpecificSNPs_10
 2195  less -S AfricanSpecificSNPs_10
 2196  ls -l *pl
 2197  cp  ChinaSNPstart2.pl  AfricaSNPstart2.pl
 2198  vi   AfricaSNPstart2.pl
 2199  perl   AfricaSNPstart2.pl
 2200  ls
 2201  wc AfricanSpecificSNPs_1
 2202  wc AfricanSpecificSNPs_2
```

```
 2203  wc AfricanSpecificSNPs_3
 2204  wc AfricanSpecificSNPs_4
 2205  wc AfricanSpecificSNPs_10
 2206  ls -l *pl
 2207  cp AmericanSNPsESTONIA.pl AfricanSNPsESTONIA.pl
 2208  vi AfricanSNPsESTONIA.pl
 2209  perl AfricanSNPsESTONIA.pl 10
 2210  ls
 2211  wc AfricanSpecificSNPsEstonia_10
 2212  less -S  AfricanSpecificSNPsEstonia_10
 2213  ls -l *pl
 2214  cp AmericanSNPstart3.pl AfricanSNPstart3.pl
 2215  vi  AfricanSNPstart3.pl
 2216  perl  AfricanSNPstart3.pl
```

Ok. I finished AFR calculations. At this stage it is impossible to compare AFR, OCE, and AMR results because they have different thresholds for allele frequencies to be region-specific. It starts from Step1 for Simons calculations. The number of indiviudals from AFR, OCE, and AMR is different and their purity may vary. For Africans I chose 22 counts for 47 AFR Simon individuals, which is pretty high. At the end I got 31,943 African-specific SNPs.

```
afedorov@bio:~/NOV2021$ wc AfricanSpecificSNPsEstonia_1
  2382  40454 189798 AfricanSpecificSNPsEstonia_1
afedorov@bio:~/NOV2021$ wc AfricanSpecificSNPsEstonia_2
  2863  48374 226793 AfricanSpecificSNPsEstonia_2
afedorov@bio:~/NOV2021$ wc AfricanSpecificSNPsEstonia_3
  2549  42892 199679 AfricanSpecificSNPsEstonia_3
afedorov@bio:~/NOV2021$ wc AfricanSpecificSNPsEstonia_4
  2206  37154 173399 AfricanSpecificSNPsEstonia_4
afedorov@bio:~/NOV2021$ wc AfricanSpecificSNPsEstonia_5
  2265  38545 181084 AfricanSpecificSNPsEstonia_5
afedorov@bio:~/NOV2021$ wc AfricanSpecificSNPsEstonia_6
  1430  24206 112818 AfricanSpecificSNPsEstonia_6
afedorov@bio:~/NOV2021$ wc AfricanSpecificSNPsEstonia_7
  1654  27987 131041 AfricanSpecificSNPsEstonia_7
afedorov@bio:~/NOV2021$ wc AfricanSpecificSNPsEstonia_8
  2173  36953 173368 AfricanSpecificSNPsEstonia_8
afedorov@bio:~/NOV2021$ wc AfricanSpecificSNPsEstonia_9
  1574  26694 124709 AfricanSpecificSNPsEstonia_9
afedorov@bio:~/NOV2021$ wc AfricanSpecificSNPsEstonia_10
  1274  21384 100533 AfricanSpecificSNPsEstonia_10
afedorov@bio:~/NOV2021$ wc AfricanSpecificSNPsEstonia_11
  1527  25620 120063 AfricanSpecificSNPsEstonia_11
afedorov@bio:~/NOV2021$ wc AfricanSpecificSNPsEstonia_12
 1220 20738 98603 AfricanSpecificSNPsEstonia_12
afedorov@bio:~/NOV2021$ wc AfricanSpecificSNPsEstonia_13
  741 12488 58756 AfricanSpecificSNPsEstonia_13
afedorov@bio:~/NOV2021$ wc AfricanSpecificSNPsEstonia_14
 1053 17753 83766 AfricanSpecificSNPsEstonia_14
afedorov@bio:~/NOV2021$ wc AfricanSpecificSNPsEstonia_15
 1084 18268 85909 AfricanSpecificSNPsEstonia_15
afedorov@bio:~/NOV2021$ wc AfricanSpecificSNPsEstonia_16
 1167 19871 93717 AfricanSpecificSNPsEstonia_16
afedorov@bio:~/NOV2021$ wc AfricanSpecificSNPsEstonia_17
  1272  21639 101730 AfricanSpecificSNPsEstonia_17
afedorov@bio:~/NOV2021$ wc AfricanSpecificSNPsEstonia_18
  723 12147 56629 AfricanSpecificSNPsEstonia_18
afedorov@bio:~/NOV2021$ wc AfricanSpecificSNPsEstonia_19
  809 13602 63760 AfricanSpecificSNPsEstonia_19
afedorov@bio:~/NOV2021$ wc AfricanSpecificSNPsEstonia_20
  815 13816 64615 AfricanSpecificSNPsEstonia_20
afedorov@bio:~/NOV2021$ wc AfricanSpecificSNPsEstonia_21
  669 11321 53298 AfricanSpecificSNPsEstonia_21
afedorov@bio:~/NOV2021$ wc AfricanSpecificSNPsEstonia_22
  493  8331 39125 AfricanSpecificSNPsEstonia_22
```

COMPARISONS of the results for AFR, AMR, CHI, OCE:

1) ~~AFR 23 counts out of 47 people: THRESHOLD 24.5%. 31.9K~~
2) AFR 17 counts out of 47 people: THRESHOLD 18.1%. 124.4K
3) AMR 3 counts out of 8 people: THRESHOLD 18.75% 20.7K
4) ~~OCE 5 counts out of 19 people: THRESHOLD 13.2% 74.4K~~
5) OCE 7 counts out of 19 people: THRESHOLD 18.4% 37.6K
6) CHI 8 counts out of 22 people: THRESHOLD 18.2% 2.9K

Geographical Region	# Specific SNPs
Americas	20,700
Oceania	37,600
Africa	124,000
East Asia	2,900

```
afedorov@bio:~/NOV2021$ wc OceaniaSpecificSNPsEstonia_1
  3203  55546 261833 OceaniaSpecificSNPsEstonia_1
afedorov@bio:~/NOV2021$ wc OceaniaSpecificSNPsEstonia_2
  2158  37173 174841 OceaniaSpecificSNPsEstonia_2
afedorov@bio:~/NOV2021$ wc OceaniaSpecificSNPsEstonia_3
  4538  78403 369980
OceaniaSpecificSNPsEstonia_3
afedorov@bio:~/NOV2021$ wc OceaniaSpecificSNPsEstonia_4
  1857  32061 150153 OceaniaSpecificSNPsEstonia_4
afedorov@bio:~/NOV2021$ wc OceaniaSpecificSNPsEstonia_5
  2156  36988 173666 OceaniaSpecificSNPsEstonia_5
afedorov@bio:~/NOV2021$ wc OceaniaSpecificSNPsEstonia_6
  3027  52514 247959 OceaniaSpecificSNPsEstonia_6
afedorov@bio:~/NOV2021$ wc OceaniaSpecificSNPsEstonia_7
  1466  25333 118605 OceaniaSpecificSNPsEstonia_7
afedorov@bio:~/NOV2021$ wc OceaniaSpecificSNPsEstonia_8
  1863  32174 150937 OceaniaSpecificSNPsEstonia_8
afedorov@bio:~/NOV2021$ wc OceaniaSpecificSNPsEstonia_9
 1154 19620 90969 OceaniaSpecificSNPsEstonia_9
afedorov@bio:~/NOV2021$ wc OceaniaSpecificSNPsEstonia_10
 1032 17780 84846 OceaniaSpecificSNPsEstonia_10
afedorov@bio:~/NOV2021$ wc OceaniaSpecificSNPsEstonia_11
  969 16641 79641 OceaniaSpecificSNPsEstonia_11
afedorov@bio:~/NOV2021$ wc OceaniaSpecificSNPsEstonia_12
  2163  37337 178779 OceaniaSpecificSNPsEstonia_12
afedorov@bio:~/NOV2021$ wc OceaniaSpecificSNPsEstonia_13
 1116 19156 91414 OceaniaSpecificSNPsEstonia_13
afedorov@bio:~/NOV2021$ wc OceaniaSpecificSNPsEstonia_14
  1763  29942 141239 OceaniaSpecificSNPsEstonia_14
afedorov@bio:~/NOV2021$ wc OceaniaSpecificSNPsEstonia_15
  1199  20910 100064 OceaniaSpecificSNPsEstonia_15
afedorov@bio:~/NOV2021$ wc OceaniaSpecificSNPsEstonia_16
  1911  32826 156041 OceaniaSpecificSNPsEstonia_16
afedorov@bio:~/NOV2021$ wc OceaniaSpecificSNPsEstonia_17
 1110 19232 91589 OceaniaSpecificSNPsEstonia_17
afedorov@bio:~/NOV2021$ wc OceaniaSpecificSNPsEstonia_18
 1147 19503 91477 OceaniaSpecificSNPsEstonia_18
afedorov@bio:~/NOV2021$ wc OceaniaSpecificSNPsEstonia_19
 1061 18190 85384 OceaniaSpecificSNPsEstonia_19
afedorov@bio:~/NOV2021$ wc OceaniaSpecificSNPsEstonia_20
 1024 17623 83430 OceaniaSpecificSNPsEstonia_20
afedorov@bio:~/NOV2021$ wc OceaniaSpecificSNPsEstonia_21
  962 16921 81287 OceaniaSpecificSNPsEstonia_21
afedorov@bio:~/NOV2021$ wc OceaniaSpecificSNPsEstonia_22
  749 13057 62701 OceaniaSpecificSNPsEstonia_22
```

Total for the new threshold is 37,625

Now I recalculated AFR for the common threshold of 18.1%

```
afedorov@bio:~/NOV2021$ wc AfricanSpecificSNPsEstonia_1
 10057 168231 781184 AfricanSpecificSNPsEstonia_1
afedorov@bio:~/NOV2021$ wc AfricanSpecificSNPsEstonia_2
 11484 191058 885057 AfricanSpecificSNPsEstonia_2
afedorov@bio:~/NOV2021$ wc AfricanSpecificSNPsEstonia_3
  9230 153540 708534 AfricanSpecificSNPsEstonia_3
afedorov@bio:~/NOV2021$ wc AfricanSpecificSNPsEstonia_4
  8483 140647 648836 AfricanSpecificSNPsEstonia_4
afedorov@bio:~/NOV2021$ wc AfricanSpecificSNPsEstonia_5
  7972 133689 621183 AfricanSpecificSNPsEstonia_5
afedorov@bio:~/NOV2021$ wc AfricanSpecificSNPsEstonia_6
  6052 101400 469127 AfricanSpecificSNPsEstonia_6
afedorov@bio:~/NOV2021$ wc AfricanSpecificSNPsEstonia_7
  6929 115097 531173 AfricanSpecificSNPsEstonia_7
afedorov@bio:~/NOV2021$ wc AfricanSpecificSNPsEstonia_8
  7691 128391 593997 AfricanSpecificSNPsEstonia_8
afedorov@bio:~/NOV2021$ wc AfricanSpecificSNPsEstonia_9
  5815  96815 446073 AfricanSpecificSNPsEstonia_9
afedorov@bio:~/NOV2021$ wc AfricanSpecificSNPsEstonia_10
  5340  88416 411760 AfricanSpecificSNPsEstonia_10
afedorov@bio:~/NOV2021$ wc AfricanSpecificSNPsEstonia_11
  5948  98437 457231 AfricanSpecificSNPsEstonia_11
afedorov@bio:~/NOV2021$ wc AfricanSpecificSNPsEstonia_12
  5435  90227 421785 AfricanSpecificSNPsEstonia_12
afedorov@bio:~/NOV2021$ wc AfricanSpecificSNPsEstonia_13
  3389  56645 264726 AfricanSpecificSNPsEstonia_13
afedorov@bio:~/NOV2021$ wc AfricanSpecificSNPsEstonia_14
  4109  68167 317654 AfricanSpecificSNPsEstonia_14
afedorov@bio:~/NOV2021$ wc AfricanSpecificSNPsEstonia_15
  4173  69393 322752 AfricanSpecificSNPsEstonia_15
afedorov@bio:~/NOV2021$ wc AfricanSpecificSNPsEstonia_16
  4804  80245 373336 AfricanSpecificSNPsEstonia_16
afedorov@bio:~/NOV2021$ wc AfricanSpecificSNPsEstonia_17
  4205  70556 328850 AfricanSpecificSNPsEstonia_17
afedorov@bio:~/NOV2021$ wc AfricanSpecificSNPsEstonia_18
  2960  49195 227447 AfricanSpecificSNPsEstonia_18
afedorov@bio:~/NOV2021$ wc AfricanSpecificSNPsEstonia_19
  2965  49412 230208 AfricanSpecificSNPsEstonia_19
afedorov@bio:~/NOV2021$ wc AfricanSpecificSNPsEstonia_20
  2922  48390 222559 AfricanSpecificSNPsEstonia_20
afedorov@bio:~/NOV2021$ wc AfricanSpecificSNPsEstonia_21
  2377  39552 183834 AfricanSpecificSNPsEstonia_21
afedorov@bio:~/NOV2021$ wc AfricanSpecificSNPsEstonia_22
  2106  34907 161567 AfricanSpecificSNPsEstonia_22
```

Total number of AFR-specific SNPs is 124,446

November 8, 2021

Calculation of China-specific SNPs via 1000G only

HISTORY:

```
vi PopChiSNPstart.pl
 2137  perl PopChiSNPstart.pl
 2138  ls -l no*
 2139  wc no*
 2140  top
 2141  ls -l Pop*
 2142  more PopulationSpecificSNPsCHI180_15
 2143  sort -k4 -n PopulationSpecificSNPsCHI180_15
 2144  wc PopulationSpecificSNPsCHI180_15
 2145  top
```

```
 2146  ls
 2147  vi AmericanSNPs2020.pl
 2148  pwd
 2149  vi AmericanSNPstart1.pl
 2150  perl AmericanSNPstart1.pl
 2151  top
 2152  ls
 2153  vi AmericanSNPs1000G.pl
 2154  vi AmericanSNPstart2.pl
 2155  top
 2156  perl AmericanSNPstart2.pl
 2157  top
 2158  ls
 2159  viAmericanSNPsESTONIA.pl
 2160  vi AmericanSNPsESTONIA.pl
 2161  ls
 2162  vi AmericanSNPstart3.pl
 2163  top
 2164  perl AmericanSNPstart3.pl
 2165  top
 2166  ls -l Pop*
 2167  top
 2168  ls
 2169  ls -l AmericaSpecificSNPsEstonia2*
 2170  less -S AmericaSpecificSNPsEstonia2_1
 2171  less -S AmericaSpecificSNPsEstonia2_2
 2172  sort -k1 -n AmericaSpecificSNPsEstonia2_2
 2173  less -S AmericaSpecificSNPsEstonia2_2
 2174  sort -k2 -n AmericaSpecificSNPsEstonia2_2 |less -S
 2175  ls AmericaSpecificSNPsEstonia2*
 2176  ls AmericaSpecificSNPsEstonia2* >AmericaSpecificAll_Nov8
 2177  vi AmericaSpecificAll_Nov8
2203  cat PopulationSpecificSNPsCHI180_1 PopulationSpecificSNPsCHI180_2
PopulationSpecificSNPsCHI180_3 PopulationSpecificSNPsCHI180_4 PopulationSpecificSNPsCHI180_5
PopulationSpecificSNPsCHI180_6 PopulationSpecificSNPsCHI180_7 PopulationSpecificSNPsCHI180_8
PopulationSpecificSNPsCHI180_9 PopulationSpecificSNPsCHI180_10 PopulationSpecificSNPsCHI180_11
PopulationSpecificSNPsCHI180_12 PopulationSpecificSNPsCHI180_13 PopulationSpecificSNPsCHI180_14
PopulationSpecificSNPsCHI180_15 PopulationSpecificSNPsCHI180_16 PopulationSpecificSNPsCHI180_17
PopulationSpecificSNPsCHI180_18 PopulationSpecificSNPsCHI180_19 PopulationSpecificSNPsCHI180_20
PopulationSpecificSNPsCHI180_21 PopulationSpecificSNPsCHI180_22 >
PopulationSpecificSNPsCHI180_ALL
 2204  sort -k4 -n PopulationSpecificSNPsCHI180_ALL
 2205  wc PopulationSpecificSNPsCHI180_ALL
```

```
afedorov@bio:~/NOV2021$ wc PopulationSpecificSNPsCHI180_ALL
        2427 16923 81881 PopulationSpecificSNPsCHI180_ALL
```

The threshold for CHI-specific was 18% (180 counts among 5 EAS populations)
The latest China-specific was 2,427 similar to 2.9K in the previous calculations on Simon, Estonia and 1000G.

The problem for AMR-specific is that we have a few pure Native American people (8 in total if (/Cha|Kar|Sur|Pia/))

Then we have 14 admixed Native Americans (/May|Mix|Pim|Que|Zap/)) average 72% AMR genetic background, which is equivalent to 10 pure Native American genomes.
We may put more restrictions (for example: 4>= counts of the AMR specific SNPs for the second group)

In the new program purgeNativeAmericanSNPs.pl, I put the restrictions for group1 + group2 >=7 (corresponds to 19%), Plus, I add that AMR counts in 1000G >20.

With these restrictions I got 3152 AMR-specific SNPs

```
#!/usr/local/perl
$c=$c2=0;
open(IN, "AmericanSpecificALL_Nov8") ||  die "Can't open OUTPUT : $!\n";
while(<IN>) {
        chomp($_);
        @b=split(/\t/, $_);
        $POS{$b[5]}=1;
        if ((($b[1]+ $b[2])>=7 && $b[10]>=20 ) {
                                print "$b[1]\t$b[2]\t$b[10]\n";
                $c++;
        }
        if ($b[10] <4) {$c2++;
                #print "$_\n"; #here I got ~150 SNPs absent in 1000G (no RS-ids)
        }
}
print "$c\t$c2 \n";
```

November 11, 2021

Verification of the results:

CHI-specific SNPs:

```
afedorov@bio:~/NOV2021$ sort -k4 -n PopulationSpecificSNPsCHI180_ALL
…..
rs5758520   2     0     390   0     24    42356482
rs74616308  2     0     390   0     24    42365682
rs76493153  2     3     390   0     21    89719120
rs78751799  2     3     390   0     21    89721655
rs79681023  2     3     390   0     21    89721667
rs10428005  2     0     391   0     20    42441213
rs5751201   2     0     391   0     22    42410842
rs5758554   2     0     391   0     20    42434621
rs78809963  2     3     391   0     21    89726336
rs76607656  2     3     392   0     21    89723704
rs79802711  1     5     418   0     21    133043841
rs76470826  0     1     428   0     5     28216972
rs1800414   0     0     429   0     4     28197037
rs76930569  0     0     429   0     4     28196145
rs75295597  0     0     430   0     9     28227926
rs78456448  0     0     447   0     0     100080311
rs75721934  0     0     451   0     1     100142780
(see Ensemble browser screenshot below for the last highlighted SNP
```

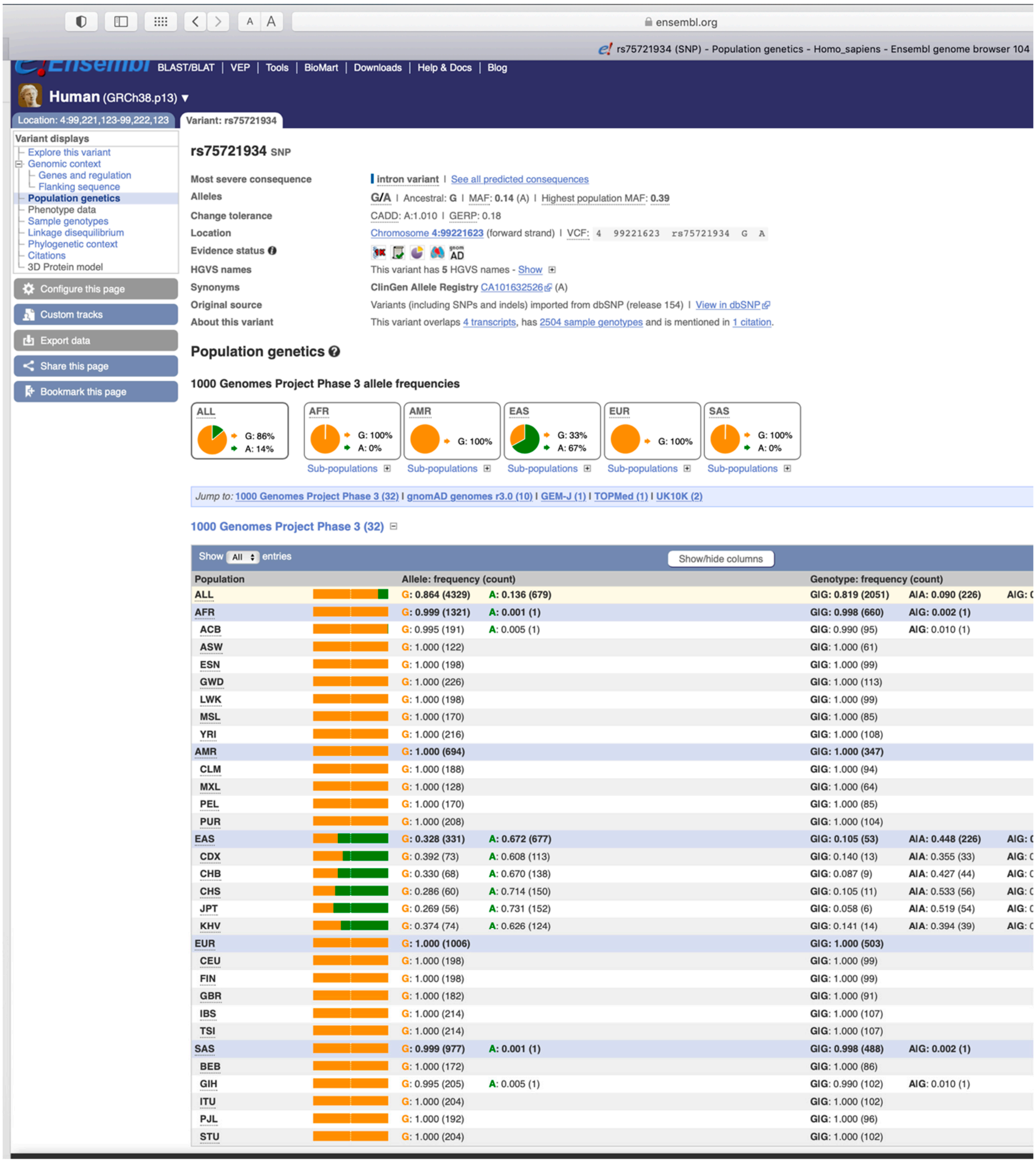

rs75721934 SNP

Most severe consequence	intron variant I See all predicted consequences
Alleles	G/A I Ancestral: G I MAF: 0.14 (A) I Highest population MAF: 0.39
Change tolerance	CADD: A:1.010 I GERP: 0.18
Location	Chromosome 4:99221623 (forward strand) I VCF: 4 99221623 rs75721934 G A
Evidence status	
HGVS names	This variant has 5 HGVS names - Show
Synonyms	ClinGen Allele Registry CA101632526 (A)
Original source	Variants (including SNPs and indels) imported from dbSNP (release 154) I View in dbSNP
About this variant	This variant overlaps 4 transcripts, has 2504 sample genotypes and is mentioned in 1 citation.

Population genetics

1000 Genomes Project Phase 3 allele frequencies

ALL G: 86% A: 14% | AFR G: 100% A: 0% | AMR G: 100% | EAS G: 33% A: 67% | EUR G: 100% | SAS G: 100% A: 0%

Jump to: 1000 Genomes Project Phase 3 (32) I gnomAD genomes r3.0 (10) I GEM-J (1) I TOPMed (1) I UK10K (2)

1000 Genomes Project Phase 3 (32)

Population	Allele: frequency (count)		Genotype: frequency (count)		
ALL	G: 0.864 (4329)	A: 0.136 (679)	G|G: 0.819 (2051)	A|A: 0.090 (226)	A|G: [illegible]
AFR	G: 0.999 (1321)	A: 0.001 (1)	G|G: 0.998 (660)	A|G: 0.002 (1)	
ACB	G: 0.995 (191)	A: 0.005 (1)	G|G: 0.990 (95)	A|G: 0.010 (1)	
ASW	G: 1.000 (122)		G|G: 1.000 (61)		
ESN	G: 1.000 (198)		G|G: 1.000 (99)		
GWD	G: 1.000 (226)		G|G: 1.000 (113)		
LWK	G: 1.000 (198)		G|G: 1.000 (99)		
MSL	G: 1.000 (170)		G|G: 1.000 (85)		
YRI	G: 1.000 (216)		G|G: 1.000 (108)		
AMR	G: 1.000 (694)		G|G: 1.000 (347)		
CLM	G: 1.000 (188)		G|G: 1.000 (94)		
MXL	G: 1.000 (128)		G|G: 1.000 (64)		
PEL	G: 1.000 (170)		G|G: 1.000 (85)		
PUR	G: 1.000 (208)		G|G: 1.000 (104)		
EAS	G: 0.328 (331)	A: 0.672 (677)	G|G: 0.105 (53)	A|A: 0.448 (226)	A|G: [illegible]
CDX	G: 0.392 (73)	A: 0.608 (113)	G|G: 0.140 (13)	A|A: 0.355 (33)	A|G: [illegible]
CHB	G: 0.330 (68)	A: 0.670 (138)	G|G: 0.087 (9)	A|A: 0.427 (44)	A|G: [illegible]
CHS	G: 0.286 (60)	A: 0.714 (150)	G|G: 0.105 (11)	A|A: 0.533 (56)	A|G: [illegible]
JPT	G: 0.269 (56)	A: 0.731 (152)	G|G: 0.058 (6)	A|A: 0.519 (54)	A|G: [illegible]
KHV	G: 0.374 (74)	A: 0.626 (124)	G|G: 0.141 (14)	A|A: 0.394 (39)	A|G: [illegible]
EUR	G: 1.000 (1006)		G|G: 1.000 (503)		
CEU	G: 1.000 (198)		G|G: 1.000 (99)		
FIN	G: 1.000 (198)		G|G: 1.000 (99)		
GBR	G: 1.000 (182)		G|G: 1.000 (91)		
IBS	G: 1.000 (214)		G|G: 1.000 (107)		
TSI	G: 1.000 (214)		G|G: 1.000 (107)		
SAS	G: 0.999 (977)	A: 0.001 (1)	G|G: 0.998 (488)	A|G: 0.002 (1)	
BEB	G: 1.000 (172)		G|G: 1.000 (86)		
GIH	G: 0.995 (205)	A: 0.005 (1)	G|G: 0.990 (102)	A|G: 0.010 (1)	
ITU	G: 1.000 (204)		G|G: 1.000 (102)		
PJL	G: 1.000 (192)		G|G: 1.000 (96)		
STU	G: 1.000 (204)		G|G: 1.000 (102)		

Conclusion: I verified ~ 10 random CHI-specific SNPs with Ensemble, and all of them are EAS-specific. The frequencies in Ensemble were always >10% higher than my calculations. For example for this `rs75721934` SNP in the picture, my frequency is 451 / (431 x2) =52.3%. I also took into account non-perfect EAS ancestry by calculation adjustment:

JPT 104 individuals 90% = 93.6 adjusted individuals

KHV 99 x 81% =79.2 inds

CDX 93 x 85% =79.9

CHB 103 x 86% = 88.6
CHS 105 x 0.87% = 91.3
Sum of EAS adjusted individuals is 431

I think that Ensemble uses more recent versions of 1000G that have more SNPs compared to my phase3 from ~ 5 years ago.

AMR-specific SNPs verification:

```
afedorov@bio:~/NOV2021$  sort -k2 -n AmericanSpecificALL_Nov8 |less -S
```

I sorted the AMR-specific SNPs :

```
rs149138531  10  3   1  0  CHR3   43692169   rs149138531  C  A  59   0  0  1  0  PUR  PUR  PUR  PUR
rs150420755  10  4   1  0  CHR3   43765460   rs150420755  T  C  56   0  0  1  0  PUR  PUR  CLM  CLM
rs181969062  10  4   0  0  CHR5   139927228  rs181969062  C  G  53   0  0  0  0  PUR  PUR  PUR  PUR
rs182746236  10  2   1  0  CHR8   144578359  rs182746236  C  T  55   0  0  0  0  PUR  PUR  CLM  CLM
rs185167736  10  0   0  0  CHR13  109018802  rs185167736  A  G  24   0  0  0  0  CLM  CLM  CLM  PUR
rs186957903  10  2   1  0  CHR8   144563585  rs186957903  C  T  53   0  0  0  0  PUR  PUR  CLM  CLM
rs187933875  10  3   0  0  CHR2   55113738   rs187933875  G  A  39   0  0  0  0  PUR  PUR  PUR  PUR
rs188012763  10  1   0  0  CHR12  102309487  rs188012763  G  A  11   0  1  0  0  PUR  PUR  CLM  CLM
rs188143812  10  1   1  0  CHR3   4943516 rs188143812     G  T  31   0  1  0  1  PUR  PUR  PUR  PUR  PUR
rs189392845  10  5   0  0  CHR13  34004204   rs189392845  G  A  39   0  1  0  0  PUR  PUR  PUR  PUR
rs189980011  10  8   0  0  CHR1   39422598   rs189980011  C  T  76   0  0  0  0  PUR  PUR  CLM  CLM
rs192181860  10  4   0  0  CHR11  114588103  rs192181860  C  T  41   0  0  0  0  PUR  PUR  CLM  CLM
rs192192450  10  2   1  1  CHR6   94784299   rs192192450  G  A  40   0  1  0  0  PUR  PUR  PUR  PUR
rs192854042  10  8   0  0  CHR1   39415060   rs192854042  C  G  75   0  0  0  0  PUR  PUR  CLM  CLM
rs4861299    10  4   0  0  CHR4   40335023   rs4861299    C  T  46   0  0  0  0  CLM  CLM  CLM  PUR
rs12476847   11  12  1  0  CHR2   164002001  rs12476847   C  G  114  1  0  1  0  PUR  PUR  PUR  PUR
rs138262411  11  8   0  0  CHR5   139193402  rs138262411  G  A  54   0  0  0  0  PUR  PUR  PUR  PUR
rs140471724  11  5   0  0  CHR8   108820970  rs140471724  G  A  39   0  0  0  0  PUR  PUR  PUR  PUR
rs141251427  11  2   0  0  CHR1   80866681   rs141251427  A  G  26   0  0  0  0  CLM  CLM  CLM  CLM
rs145730875  11  1   0  0  CHR8   51993585   rs145730875  T  C  36   0  0  0  0  PUR  PUR  PUR  PUR
rs12453180   12  5   0  0  CHR17  77707453   rs12453180   T  C  59   0  0  0  0  PUR  PUR  PUR  CLM
rs138541449  12  14  1  0  CHR2   164088448  rs138541449  T  G  116  0  0  1  0  PUR  PUR  PUR  PUR
rs139100995  12  4   3  0  CHR13  101295050  rs139100995  C  T  74   0  0  0  0  PUR  PUR  PUR  PUR
rs150480779  12  4   0  0  CHR5   139636352  rs150480779  C  T  54   1  0  0  0  FIN  PUR  PUR  PUR
rs189842535  13  4   0  0  CHR10  85801823   rs189842535  T  G  31   0  0  0  0  PUR  PUR  PUR  PUR
rs139106598  14  9   1  0  CHR12  75253681   rs139106598  T  C  50   1  0  0  0  GBR  PUR  PUR  PUR
(END)
```

Below is the Ensemble verification of the last SNP rs139106598:
The frequency in 1000G is low ~7% because of the huge admixture in AMR populations.
The columns and 3 shows counts in Simons' project and they are > 50%.

All in all, the AMR-specific SNPs are true!

rs139106598 SNP

Most severe consequence	intergenic variant
Alleles	T/C | Ancestral: T | MAF: 0.01 (C) | Highest population MAF: 0.13
Change tolerance	CADD: C:2.751 | GERP: -1.78
Location	Chromosome 12:74859901 (forward strand) | VCF: `12 74859901 rs139106598 T C`
Evidence status	gnom AD
HGVS name	NC_000012.12:g.74859901T>C
Synonyms	ClinGen Allele Registry CA239857161 (C)
Original source	Variants (including SNPs and indels) imported from dbSNP (release 154) | View in dbSNP
About this variant	This variant has 2504 sample genotypes.

Population genetics

1000 Genomes Project Phase 3 allele frequencies

ALL	AFR	AMR	EAS	EUR	SAS
T: 99% C: 1%	T: 100% C: 0%	T: 92% C: 8%	T: 100%	T: 100% C: 0%	T: 100%
	Sub-populations	Sub-populations	Sub-populations	Sub-populations	Sub-populations

Jump to: 1000 Genomes Project Phase 3 (32) | gnomAD genomes r3.0 (10) | NCBI ALFA (10) | TOPMed (1)

1000 Genomes Project Phase 3 (32)

Show All entries   Show/hide columns

Population	Allele: frequency (count)		Genotype: freque
ALL	T: 0.989 (4952)	C: 0.011 (56)	T|T: 0.979 (2452)
AFR	T: 0.999 (1321)	C: 0.001 (1)	T|T: 0.998 (660)
ACB	T: 1.000 (192)		T|T: 1.000 (96)
ASW	T: 0.992 (121)	C: 0.008 (1)	T|T: 0.984 (60)
ESN	T: 1.000 (198)		T|T: 1.000 (99)
GWD	T: 1.000 (226)		T|T: 1.000 (113)
LWK	T: 1.000 (198)		T|T: 1.000 (99)
MSL	T: 1.000 (170)		T|T: 1.000 (85)
YRI	T: 1.000 (216)		T|T: 1.000 (108)
AMR	T: 0.922 (640)	C: 0.078 (54)	T|T: 0.856 (297)
CLM	T: 0.936 (176)	C: 0.064 (12)	T|T: 0.883 (83)
MXL	T: 0.938 (120)	C: 0.062 (8)	T|T: 0.875 (56)
PEL	T: 0.871 (148)	C: 0.129 (22)	T|T: 0.753 (64)
PUR	T: 0.942 (196)	C: 0.058 (12)	T|T: 0.904 (94)
EAS	T: 1.000 (1008)		T|T: 1.000 (504)
CDX	T: 1.000 (186)		T|T: 1.000 (93)
CHB	T: 1.000 (206)		T|T: 1.000 (103)
CHS	T: 1.000 (210)		T|T: 1.000 (105)
JPT	T: 1.000 (208)		T|T: 1.000 (104)
KHV	T: 1.000 (198)		T|T: 1.000 (99)
EUR	T: 0.999 (1005)	C: 0.001 (1)	T|T: 0.998 (502)
CEU	T: 1.000 (198)		T|T: 1.000 (99)
FIN	T: 1.000 (198)		T|T: 1.000 (99)
GBR	T: 0.995 (181)	C: 0.005 (1)	T|T: 0.989 (90)
IBS	T: 1.000 (214)		T|T: 1.000 (107)
TSI	T: 1.000 (214)		T|T: 1.000 ([illegible])

Now OCE-specific SNPs:

`more OceaniaSpecificSNPsEstonia2_ALL`
They are practically absent in 1000G by obvious reasons (no OCE among 1000G)
All in all, the results are good!

November 12, 2021

AFR-specific

Finished.

November 13, 2021

Examination of the reverse situation: In EAS allele is absent, while in other region this allele is frequent (counting 0 instead of 1 in VCF). For this purpose I created two programs: `PopulationSpecific1000gCHIreverse.pl` and `ChinaSNPstart2reverse.pl`. It works on 1000G dataset.

Below is the example of one of such SNPs. In total for EAS I got 102 of such SNPs, some of them are indels.

```
afedorov@bio:~/NOV2021$ wc PopulationSpecificSNPsCHI180reverse_ALL
 102  816 4839 PopulationSpecificSNPsCHI180reverse_ALL
```

My interpretation is that one of the frequent alleles has been fixed in EAS, while its frequent counterpart allele is all over the World except EAS.

It takes about 30 min to run this program on Chr22. And 3-4 hours for the entire genome.

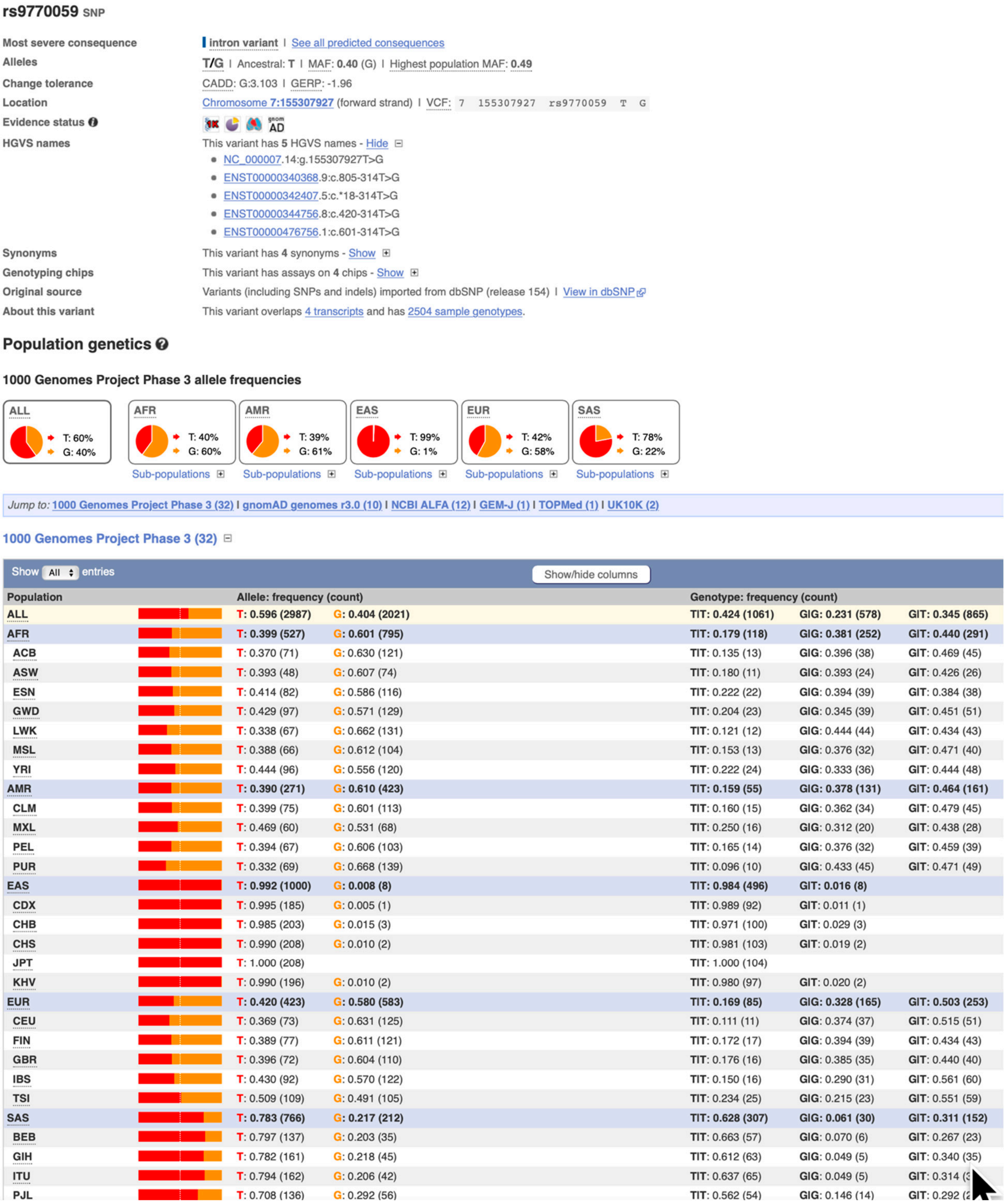

rs9770059 SNP

Most severe consequence	intron variant | See all predicted consequences
Alleles	T/G | Ancestral: T | MAF: 0.40 (G) | Highest population MAF: 0.49
Change tolerance	CADD: G:3.103 | GERP: -1.96
Location	Chromosome 7:155307927 (forward strand) | VCF: `7 155307927 rs9770059 T G`
Evidence status	gnom AD
HGVS names	This variant has 5 HGVS names - Hide<br>• NC_000007.14:g.155307927T>G<br>• ENST00000340368.9:c.805-314T>G<br>• ENST00000342407.5:c.*18-314T>G<br>• ENST00000344756.8:c.420-314T>G<br>• ENST00000476756.1:c.601-314T>G
Synonyms	This variant has 4 synonyms - Show
Genotyping chips	This variant has assays on 4 chips - Show
Original source	Variants (including SNPs and indels) imported from dbSNP (release 154) | View in dbSNP
About this variant	This variant overlaps 4 transcripts and has 2504 sample genotypes.

Population genetics

1000 Genomes Project Phase 3 allele frequencies

ALL	AFR	AMR	EAS	EUR	SAS
T: 60%<br>G: 40%	T: 40%<br>G: 60%	T: 39%<br>G: 61%	T: 99%<br>G: 1%	T: 42%<br>G: 58%	T: 78%<br>G: 22%
	Sub-populations	Sub-populations	Sub-populations	Sub-populations	Sub-populations

Jump to: 1000 Genomes Project Phase 3 (32) I gnomAD genomes r3.0 (10) I NCBI ALFA (12) I GEM-J (1) I TOPMed (1) I UK10K (2)

1000 Genomes Project Phase 3 (32)

Show All entries   Show/hide columns

Population	Allele: frequency (count)		Genotype: frequency (count)		
**ALL**	**T: 0.596 (2987)**	**G: 0.404 (2021)**	**TIT: 0.424 (1061)**	**GIG: 0.231 (578)**	**GIT: 0.345 (865)**
**AFR**	**T: 0.399 (527)**	**G: 0.601 (795)**	**TIT: 0.179 (118)**	**GIG: 0.381 (252)**	**GIT: 0.440 (291)**
ACB	T: 0.370 (71)	G: 0.630 (121)	TIT: 0.135 (13)	GIG: 0.396 (38)	GIT: 0.469 (45)
ASW	T: 0.393 (48)	G: 0.607 (74)	TIT: 0.180 (11)	GIG: 0.393 (24)	GIT: 0.426 (26)
ESN	T: 0.414 (82)	G: 0.586 (116)	TIT: 0.222 (22)	GIG: 0.394 (39)	GIT: 0.384 (38)
GWD	T: 0.429 (97)	G: 0.571 (129)	TIT: 0.204 (23)	GIG: 0.345 (39)	GIT: 0.451 (51)
LWK	T: 0.338 (67)	G: 0.662 (131)	TIT: 0.121 (12)	GIG: 0.444 (44)	GIT: 0.434 (43)
MSL	T: 0.388 (66)	G: 0.612 (104)	TIT: 0.153 (13)	GIG: 0.376 (32)	GIT: 0.471 (40)
YRI	T: 0.444 (96)	G: 0.556 (120)	TIT: 0.222 (24)	GIG: 0.333 (36)	GIT: 0.444 (48)
**AMR**	**T: 0.390 (271)**	**G: 0.610 (423)**	**TIT: 0.159 (55)**	**GIG: 0.378 (131)**	**GIT: 0.464 (161)**
CLM	T: 0.399 (75)	G: 0.601 (113)	TIT: 0.160 (15)	GIG: 0.362 (34)	GIT: 0.479 (45)
MXL	T: 0.469 (60)	G: 0.531 (68)	TIT: 0.250 (16)	GIG: 0.312 (20)	GIT: 0.438 (28)
PEL	T: 0.394 (67)	G: 0.606 (103)	TIT: 0.165 (14)	GIG: 0.376 (32)	GIT: 0.459 (39)
PUR	T: 0.332 (69)	G: 0.668 (139)	TIT: 0.096 (10)	GIG: 0.433 (45)	GIT: 0.471 (49)
**EAS**	**T: 0.992 (1000)**	**G: 0.008 (8)**	**TIT: 0.984 (496)**	**GIT: 0.016 (8)**	
CDX	T: 0.995 (185)	G: 0.005 (1)	TIT: 0.989 (92)	GIT: 0.011 (1)	
CHB	T: 0.985 (203)	G: 0.015 (3)	TIT: 0.971 (100)	GIT: 0.029 (3)	
CHS	T: 0.990 (208)	G: 0.010 (2)	TIT: 0.981 (103)	GIT: 0.019 (2)	
JPT	T: 1.000 (208)		TIT: 1.000 (104)		
KHV	T: 0.990 (196)	G: 0.010 (2)	TIT: 0.980 (97)	GIT: 0.020 (2)	
**EUR**	**T: 0.420 (423)**	**G: 0.580 (583)**	**TIT: 0.169 (85)**	**GIG: 0.328 (165)**	**GIT: 0.503 (253)**
CEU	T: 0.369 (73)	G: 0.631 (125)	TIT: 0.111 (11)	GIG: 0.374 (37)	GIT: 0.515 (51)
FIN	T: 0.389 (77)	G: 0.611 (121)	TIT: 0.172 (17)	GIG: 0.394 (39)	GIT: 0.434 (43)
GBR	T: 0.396 (72)	G: 0.604 (110)	TIT: 0.176 (16)	GIG: 0.385 (35)	GIT: 0.440 (40)
IBS	T: 0.430 (92)	G: 0.570 (122)	TIT: 0.150 (16)	GIG: 0.290 (31)	GIT: 0.561 (60)
TSI	T: 0.509 (109)	G: 0.491 (105)	TIT: 0.234 (25)	GIG: 0.215 (23)	GIT: 0.551 (59)
**SAS**	**T: 0.783 (766)**	**G: 0.217 (212)**	**TIT: 0.628 (307)**	**GIG: 0.061 (30)**	**GIT: 0.311 (152)**
BEB	T: 0.797 (137)	G: 0.203 (35)	TIT: 0.663 (57)	GIG: 0.070 (6)	GIT: 0.267 (23)
GIH	T: 0.782 (161)	G: 0.218 (45)	TIT: 0.612 (63)	GIG: 0.049 (5)	GIT: 0.340 (35)
ITU	T: 0.794 (162)	G: 0.206 (42)	TIT: 0.637 (65)	GIG: 0.049 (5)	GIT: 0.314 ([illegible]
PJL	T: 0.708 (136)	G: 0.292 (56)	TIT: 0.562 (54)	GIG: 0.146 (14)	GIT: 0.292 ([illegible]

These 102 SNPs are enriched with triplets and indels:

```
afedorov@bio:~/NOV2021$ more PopulationSpecificSNPsCHI180reverse_ALL
rs550508207;rs61288664 189   154   6     1360  212   10250248    1915
rs537584292;rs10548470 378   209   4     1082  139   31623062    1808
rs587637054;rs113676423 380  848   4     932   467   163662893   2627
rs7552755    258   530   8     1045  144   236062563   1977
```

```
rs10189730  411   414   8     858   315   5233900     1998
rs11690918  284   348   6     926   464   43474112    2022
rs7593400   187   422   8     1174  252   71533192    2035
rs552762013;rs139573656 270     524   4     1177  294   103783737   2265
rs10200938  345   910   3     413   452   177645345   2120
rs552019620 140   352   3     1248  190   213574967   1930
rs7570666   361   932   8     379   454   216634123   2126
rs7578746   340   956   6     680   390   228546406   2366
rs567101642;rs570614020;rs563012911;rs555478859      434   873   9     929   516     6147144 2752
rs369258977 311   701   8     842   292   34214157    2146
rs77058777  244   674   2     918   120   44586890    1956
rs12715530  285   536   3     696   309   58354251    1826
rs183926141;rs540644886 396     765   2     449   336   58409536    1946
rs550762285;rs551139233 461     442   4     665   316   59135511    1884
rs561991767;rs6784685   546     604   0     1188  271   59172575    2609
rs550503162;rs11426073  182     284   2     1194  324   116952698   1984
rs560428318;rs10575001  261     640   4     755   320   141097203   1976
rs537443279;rs142683341 286     405   6     944   229   194020689   1864
rs765831    269   446   8     829   292   6033657     1836
rs355689    227   532   4     1119  206   78507797    2084
rs9993932   300   623   8     821   362   92682249    2106
rs189406292;rs56866437  310     464   3     998   186   121276184   1958
rs35384592  323   812   4     289   424   190019703   1848
rs2035612   328   630   4     454   430   1993600     1842
rs7356708   130   90    0     1467  120   130593723   1807
rs7739347   383   705   8     391   357   7026977     1836
rs9505001   409   705   9     479   362   7027384     1955
rs6904651   408   705   9     348   354   7032763     1815
rs545837318;rs139148321 683     1314  6     204   514   7060560     2715
rs531005467;rs149418128 237     267   8     1290  166   50459249    1960
rs545402591;rs2260243   275     540   6     975   364   163860387   2154
rs149006477 506   612   8     1142  409   7902650     2669
rs10231872  185   381   2     901   524   16598126    1991
rs56335835  219   394   9     994   375   20606519    1982
rs1525030   398   770   4     1320  482   108859727   2970
rs9770059   423   583   8     600   212   155099637   1818
rs9770060   423   583   8     605   212   155099650   1823
rs9770068   420   583   8     592   212   155099762   1807
rs7012429   206   302   0     1228  79    2967970     1815
rs149646093 339   826   6     543   387   8925433     2095
rs6601427   270   581   6     651   384   10156025    1886
rs533365216;rs72283782  403     1012  1     410   480   66398717    2305
rs4433240   484   1450  0     16    348   16801130    2298
rs373029613;rs7043103   373     283   6     1097  155   91664206    1908
rs551863172 344   480   4     895   234   109733656   1953
rs370175927 445   640   9     606   157   873215      1848
rs12261591  199   304   3     1516  103   17077390    2122
rs137905132 121   281   3     1153  407   31739802    1962
rs112291200;rs7904458   251     569   6     936   284   45342273    2040
rs4622198   216   564   4     925   172   78923581    1877
rs7904234   311   276   0     1227  204   109724292   2018
rs644635    267   573   2     787   258   100756968   1885
rs601496    260   570   3     732   256   100762986   1818
rs576278    259   569   2     740   250   100768790   1818
rs662870    265   571   2     773   254   100775268   1863
```

I found a bug in Step2.pl programs. It counts 1|1 as a single allele and should be as two alleles.
I updated V2 of the program:

```
2174  cp China1000gStep2.pl China1000gStep2_v2.pl
 2175  vi China1000gStep2_v2.pl
 2176  ls -l *pl
 2177  vi ChinaSNPstart2.pl
 2178  perl ChinaSNPstart2.pl (at 21:02 ->)
```

```
for $y (9..$#e) {
                                $p1=0;
```

```
                                        if ($e[$y] =~/^(\d)\|(\d)/){
                                               if ($1>1.5) {$1 =0;}
                                               if ($2>1.5) {$2 =0;}
                                               $p1 = $1 + $2;
                                        }
                                        if ($p1 >0 ) {
                                               #       print  "$column_pop{$y}\t";
                                                       $line .=$column_pop{$y} ."\t";
                                                       if($column_pop{$y} =~/CHB|CHS|CDX|JPT|KHV/)
{$asia+=$p1;}
                                                       if($column_pop{$y} =~/GBR|FIN|CEU|IBS|TSI/)
{$eur+=$p1;}
                                                       if($column_pop{$y} =~/YRI|LWK|GWD|MSL|ESN|ACB/)
{$afr+=$p1;}
                                                       if($column_pop{$y} =~/GIH|PJL|BEB|STU|ITU/)
{$ind+=$p1;}
                                                       if($column_pop{$y} =~/MXL|PUR|CLM|PEL/)
{$amr+=$p1;}
                                                       #       print
"$column_pop{$y}_$column_cont{$y}_$column_id{$y}\t";
                                        }
                                }
```

November 24, 2021

Fixing the previous problem with Step2 programs for AFR, AMR, OCE.
HISTORY:

```
cd NOV2021/
 3331  ls
 3332  ls -l *pl
 3333  vi China1000gStep2_v2.pl
 3334  head -75 China1000gStep2_v2.pl |tail -20
 3335  ls -l *pl |grep Step2
 3336  cp Africa1000gStep2.pl Africa1000gStep2_v2.pl
 3337  vi Africa1000gStep2_v2.pl
 3338  cp AmericanSNPs1000G.pl America1000gStep2_v2.pl
 3339  vi America1000gStep2_v2.pl
 3340  vi Oceania1000gStep2new.pl
 3341  vi Oceania1000gStep2new.pl
 3342  cp Oceania1000gStep2new.pl Oceania1000gStep2new_v2.pl
 3343  ls -l *pl |grep Step2
 3344  rm Oceania1000gStep2new.pl
 3345  ls -l *pl |grep Step2
 3346  ls -l *pl
 3347  vi ChinaSNPstart2.pl
 3348  vi AfricaSNPstart2.pl
 3349  vi AmericanSNPstart2.pl
 3350  vi OceaniaSNPstart2.pl
 3351  perl  AfricaSNPstart2.pl
```

I started AfricaSNPstart2.pl at 19:10   19:58 (has been finished)

AfricaSNPstart3.pl took about 20 min.

I finished recalculation of AFR, AMR, CHI, OCE. Below is history:

```
3351  perl  AfricaSNPstart2.pl
 3352  history
 3353  top
 3354  ls
 3355  ls -l *pl
 3356  perl  AfricanSNPstart3.pl
 3357  top
 3358  ls
 3359  ls -l *pl
```

```
3360  perl  AmericanSNPstart2.pl
3361  top
3362  ls
3363  ls -l *pl
3364  perl  AmericanSNPstart3.pl
3365  top
3366  ls
3367  ls -l *pl
3368  vi OceaniaSNPstart2.pl
3369  perl OceaniaSNPstart2.pl
3370  top
3371  perl OceaniaSNPstart3.pl
3372  top
3373  ls -l *pl
3374  vi ChinaSNPstart2
3375  vi ChinaSNPstart2.pl
3376  vi  ChinaSNPstart2reverse.pl
3377  perl ChinaSNPstart2.pl
3378  top
3379  ls -l *pl
3380  perl  ChinaSNPstart3.pl
```

March 15, 2022

SUMMARY ON OCE-specific SNP dataset

1) Start from Simons' dataset. The program counts number of Alt-allele SNPs in OCE: /Aus|Bou|Pap/, which should be 7 or more alleles (among 19 people) The program also counts this allele in OCE-flanking populations: /Dus|Mao|Igo|Hav/. In the rest of Simons' populations this allele must be absent. It creates the phase-1 OCE-specific SNPs (OCE_SNPs_*$chr*), where $chr means the number of chromosome (from 1 to 22)

2) Comparison with 1000G dataset. The second program compares phase-1 OCE-specific SNPs with all 2504 individuals from 1000G. The number of "OCE-specific phase-1" alleles in all 1000G should be less or equal to the number of these alleles among 19 OCE people. Thus, the frequency of OCE-specific SNPs at this second step must be >100 times frequent than in the rest of the world. This computation creates phase-2 OCE-specific SNPs, which are stored in the OceaniaSpecificSNPs_*$chr* files.

3) Final comparison with Estonian dataset using OceanEstoniaStep3.pl program. The number of OCE-specific alleles among all Estonian DB people must be 4 or less. This is the final phase-3 purification. The main results are in the files: OceaniaSpecificSNPsEstonia2_*$chr.* This file contains the number of OCE-specific alleles in all datasets (Simons, 1000G, and Estonian) and also the name of populations, where they present.

```
rs190585583     8       0       0       0       CHR1    769040  rs190585583     A       T       0       0       0
0       0       NOT
rs373000721     9       0       0       0       CHR1    833529  rs373000721     G       A       0       0       0
0       0       NOT     Koi_OCE Kos_OCE Koi_OCE
rs367861531     10      0       0       0       CHR1    835831  rs367861531     G       A       0       0       0
1       0       YRI             Koi_OCE Kos_OCE Koi_OCE
rs369581566     10      0       0       0       CHR1    837238  rs369581566     G       A       0       0       0
0       0       NOT     Koi_OCE Kos_OCE Koi_OCE
rs377052638     8       0       0       0       CHR1    837992  rs377052638     C       G       0       0       0
0       0       NOT     Koi_OCE Kos_OCE Koi_OCE
rs373451994     10      0       0       0       CHR1    839636  rs373451994     G       A       0       0       0
0       0       NOT     Koi_OCE Kos_OCE Koi_OCE
rs146441147     9       0       0       0       CHR1    979559  rs146441147     C       T       0       0       0
0       0       NOT     Kos_OCE Koi_OCE
rs372205897     12      0       0       0       CHR1    1029701 rs372205897     C       T       0       0       0
0       0       NOT     Kos_OCE Koi_OCE
rs373322116     12      0       0       0       CHR1    1047802 rs373322116     G       A       0       0       0
0       0       NOT     Kos_OCE Koi_OCE
```

March 25, 2022

New thresholds for Region-Specific SNPs.

EUR- specific:

1) >18% for Simon project

Region/Populations	#SNPs Region-specific	#SNPs Region-specific	#SNPs Region-specific	
	Step-1; Simons' DB	V2: Step-2; 1000 Genomes	V3: Step-2; 1000 Genomes	
Africa	204,983	157,492	112,658	
Europe	6,585	3,952	1,539	
East Asia	7,789	2,893	441	
Native America	46,994	20,754	4,133	
Oceania	77,437	73,434	71,848	

SEE ANOTHER DOCUMENT: OCEspecificSNPtable.docx

March 28, 2022

Protocols from History:

```
2629  cp ChinaSNPsESTONIA_v3.pl EuropeSNPsESTONIA_v3.pl
 2630  vi EuropeSNPsESTONIA_v3.pl
 2631  vi EuropeSNPstart3.pl
 2632  perl EuropeSNPstart3.pl
 2633  top
 2634  ls
 2635  more   AmericaSpecificEstoniaV3_2_1
 2636  less -S   AmericaSpecificEstoniaV3_2_1
 2637  ls
 2638  top
 2639  ls
 2640  wc    EuropeSpecificEstoniaV3_2_1
 2641  wc    EuropeSpecificEstoniaV3_2_2
 2642  wc    EuropeSpecificEstoniaV3_2_3
 2643  wc    EuropeSpecificEstoniaV3_2_4
 2644  wc    EuropeSpecificEstoniaV3_2_5
 2645  wc    EuropeSpecificEstoniaV3_2_6
```

```
2646  wc    EuropeSpecificEstoniaV3_2_7
2647  wc    EuropeSpecificEstoniaV3_2_8
2648  wc    EuropeSpecificEstoniaV3_2_9
2649  wc    EuropeSpecificEstoniaV3_2_10
2650  wc    EuropeSpecificEstoniaV3_2_11
2651  wc    EuropeSpecificEstoniaV3_2_12
2652  wc    EuropeSpecificEstoniaV3_2_13
2653  wc    EuropeSpecificEstoniaV3_2_14
2654  wc    EuropeSpecificEstoniaV3_2_15
2655  wc    EuropeSpecificEstoniaV3_2_16
2656  wc    EuropeSpecificEstoniaV3_2_17
2657  wc    EuropeSpecificEstoniaV3_2_18
2658  wc    EuropeSpecificEstoniaV3_2_19
2659  wc    EuropeSpecificEstoniaV3_2_20
2660  wc    EuropeSpecificEstoniaV3_2_21
2661  wc    EuropeSpecificEstoniaV3_2_22
2662  history
2663  vi EuropeSNPsESTONIA_v3.pl
2664  ls
2665  wc    EuropeSpecificEstoniaV3_2_22
2666  wc EuropeSpecificV3_1
2667  wc    EuropeSpecificEstoniaV3_2_1
2668  wc    EuropeSpecificEstoniaV3_2_2
2669  wc EuropeSpecificV3_2
2670  wc EuropeSpecificV3_1
2671  wc EuropeSpecificV3_2
2672  wc EuropeSpecificV3_3
2673  wc EuropeSpecificV3_4
2674  wc EuropeSpecificV3_5
2675  wc EuropeSpecificV3_6
2676  wc EuropeSpecificV3_7
2677  wc EuropeSpecificV3_8
2678  wc EuropeSpecificV3_9
2679  wc EuropeSpecificV3_10
2680  wc EuropeSpecificV3_11
2681  wc EuropeSpecificV3_12
2682  wc EuropeSpecificV3_13
2683  wc EuropeSpecificV3_14
2684  wc EuropeSpecificV3_15
2685  wc EuropeSpecificV3_16
2686  wc EuropeSpecificV3_17
2687  wc EuropeSpecificV3_18
2688  wc EuropeSpecificV3_19
2689  wc EuropeSpecificV3_20
2690  wc EuropeSpecificV3_21
2691  wc EuropeSpecificV3_22
2692  ls -l *pl
2693  cp  EuropeSNPsESTONIA_v3.pl  AfricaSNPsESTONIA_v3.pl
2694  vi AfricaSNPsESTONIA_v3.pl
2695  history
2696  grep EUR  estonian_pops2_A2.txt |wc
2697  grep AFR  estonian_pops2_A2.txt |wc
2698  grep AFR  estonian_pops2_A2.txt
2699  vi AfricaSNPsESTONIA_v3.pl
2700  ls -l *pl
2701  vi AfricanSNPstart3.pl
2702  perl AfricanSNPstart3.pl
2703  top
2704  ls
2705  wc AfricaSpecificEstoniaV3_2_1
2706  wc AfricaSpecificEstoniaV3_2_2
2707  wc AfricaSpecificEstoniaV3_2_3
2708  wc AfricaSpecificEstoniaV3_2_4
2709  wc AfricaSpecificEstoniaV3_2_5
2710  wc AfricaSpecificEstoniaV3_2_6
2711  wc AfricaSpecificEstoniaV3_2_7
2712  wc AfricaSpecificEstoniaV3_2_8
2713  wc AfricaSpecificEstoniaV3_2_9
2714  wc AfricaSpecificEstoniaV3_2_10
2715  wc AfricaSpecificEstoniaV3_2_11
2716  wc AfricaSpecificEstoniaV3_2_12
```

```
2717  wc AfricaSpecificEstoniaV3_2_13
2718  wc AfricaSpecificEstoniaV3_2_14
2719  wc AfricaSpecificEstoniaV3_2_15
2720  wc AfricaSpecificEstoniaV3_2_16
2721  wc AfricaSpecificEstoniaV3_2_17
2722  wc AfricaSpecificEstoniaV3_2_18
2723  wc AfricaSpecificEstoniaV3_2_19
2724  wc AfricaSpecificEstoniaV3_2_20
2725  wc AfricaSpecificEstoniaV3_2_21
2726  wc AfricaSpecificEstoniaV3_2_22
2727  ls
2728  ls -l *pl
2729  cp OceaniaSNPstart3.pl ClusteringSNPs.pl
2730  vi ClusteringSNPs.pl
2731  less -S OceaniaSpecificV3_1
2732  less -S OceaniaSpecificV3_2_1
2733  less -S OceaniaSpecificEstoniaV3_2_19
2734  vi  ChinaSNPsESTONIA_v3.pl
2735  vi ClusteringSNPs.pl
2736  perl  ClusteringSNPs.pl OceaniaSpecificEstoniaV3_2_
2737  vi ClusteringSNPs.pl
2738  perl  ClusteringSNPs.pl OceaniaSpecificEstoniaV3_2_
2739  vi ClusteringSNPs.pl
2740  perl  ClusteringSNPs.pl OceaniaSpecificEstoniaV3_2_
2741  vi ClusteringSNPs.pl
2742  perl  ClusteringSNPs.pl OceaniaSpecificEstoniaV3_2_
2743  vi ClusteringSNPs.pl
2744  perl  ClusteringSNPs.pl OceaniaSpecificEstoniaV3_2_
2745  ls
2746  perl  ClusteringSNPs.pl AmericaSpecificEstoniaV3_2_
2747  perl  ClusteringSNPs.pl ChinaSpecificEstoniaV3_2_
2748  perl  ClusteringSNPs.pl EuropeSpecificEstoniaV3_2_
2749  perl  ClusteringSNPs.pl AfricaSpecificEstoniaV3_2_
2750  ls -l *pl
2751  vi  OceaniaSNPsESTONIA_v3.pl
2752  ls
2753  less -S  OceaniaSpecificEstoniaV3_2_2
2754  less -S  OceaniaSpecificEstoniaV3_2
2755  less -S  OceaniaSpecificEstoniaV3_2_2
2756  less -S  OceaniaSpecificEstoniaV3_2
2757  less -S  AfricaSpecificEstoniaV3_2
2758  ls -l *pl
2759  vi  Africa1000gStep2_v3.pl
2760  less -S  AfricaSpecificEstoniaV3_2
2761  history
```

April 9, 2022

History:

```
2017  ls -l FinalOce*
2018  less -S FinalOceania__ALL
2019  mv FinalOceania__ALL FinalOceania_ALL
2020  perl ConcatenateFiles.pl  FinalAfrica_
2021  perl ConcatenateFiles.pl  FinalEurope_
2022  perl ConcatenateFiles.pl  FinalEastAsia_
2023  perl ConcatenateFiles.pl  FinalAmerica_
2024  ls -l *ALL
2025  mv FinalAfrica__ALL FinalAfrica_ALL
2026  mv FinalAmerica__ALL FinalAmerica_ALL
2027  mv FinalEastAsia__ALL FinalEastAsia_ALL
2028  mv FinalEurope__ALL FinalEurope_ALL
2029  ls -l *ALL
2030  ls -l *pl
2031  vi  AfricanSNPstart3.pl
2032  ls
2033  ls -l *pl
2034  vi  PopulationSpecific1000gCHIreverse.pl
2035  vi  ChinaSNPstart2reverse.pl
```

```
2036  ls -l Pop*
2037  more PopulationSpecificSNPsCHI180reverse_ALL
2038  ls -l Pop*
2039  vi  PopulationSpecific1000gCHIreverse.pl
2040  cp  PopulationSpecific1000gCHIreverse.pl PopulationSpecific1000gAFRreverse.pl
2041  vi PopulationSpecific1000gAFRreverse.pl
2042  perl PopulationSpecific1000gAFRreverse.pl 22
2043  ls -l Pop*
2044  more PopulationSpecificSNPsAFR1800reverse_22
2045  vi PopulationSpecific1000gAFRreverse.pl
2046  nohup perl PopulationSpecific1000gAFRreverse.pl 22 &
2047  cp  PopulationSpecific1000gAFRreverse.pl  PopulationSpecific1000gEURreverse.pl
2048  vi PopulationSpecific1000gEURreverse.pl
2049  nuhup perl  PopulationSpecific1000gEURreverse.pl 22 &
2050  nohup perl  PopulationSpecific1000gEURreverse.pl 22 &
2051  top
2052  ls -l Pop*
2053  more PopulationSpecificSNPsAFR1800reverse_22
2054  vi PopulationSpecific1000gAFRreverse.pl
2055  history 50
2056  vi  ChinaSNPstart2reverse.pl
2057  cp  ChinaSNPstart2reverse.pl AfricaSNPstart2reverse.pl
2058  vi  AfricaSNPstart2reverse.pl
2059  rm  PopulationSpecificSNPsAFR1800reverse_22
2060  perl  AfricaSNPstart2reverse.pl
2061  vi  EuropeSNPstart2reverse.pl
2062  vi PopulationSpecific1000gEURreverse.pl
2063  perl  EuropeSNPstart2reverse.pl
2064  ls -l Pop*
2065  more PopulationSpecificSNPsAFR1500reverse_22
2066  ls -l Pop*
2067  more  PopulationSpecificSNPsEUR1000reverse_15
2068  more  PopulationSpecificSNPsAFR1500reverse_16
2069  ls -l Pop*
2070  more  PopulationSpecificSNPsEUR1000reverse_16
2071  more  PopulationSpecificSNPsEUR1000reverse_17
2072  more  PopulationSpecificSNPsEUR1000reverse_21
2073  more  PopulationSpecificSNPsEUR1000reverse_11
2074  more  PopulationSpecificSNPsAFR1500reverse_12
2075  more  PopulationSpecificSNPsAFR1500reverse_11
2076  more  PopulationSpecificSNPsAFR1500reverse_10
2077  more  PopulationSpecificSNPsAFR1500reverse_14
2078  ls -l Pop*
2079  more  PopulationSpecificSNPsCHI180reverse_3
2080  more  PopulationSpecificSNPsCHI180reverse_10
2081  more  PopulationSpecificSNPsCHI180reverse_1
2082  top
2083  ls -l Pop*
2084  more  PopulationSpecificSNPsEUR1000reverse_9
2085  more PopulationSpecificSNPsAFR1500reverse_1
2086  vi PopulationSpecific1000gEURreverse.pl
2087  history 70
2088  vi PopulationSpecific1000gAFRreverse.pl
2089  vi  PopulationSpecific1000gCHIreverse.pl
2090  ls -l Pop*pl
2091  ls -l *reverse.pl
2092  ls -l Pop*
2093  ls -l *reverse.pl
2094  nohup  AfricaSNPstart2reverse.pl &
2095  nohup  perl AfricaSNPstart2reverse.pl &
2096  nohup perl  EuropeSNPstart2reverse.pl &
2097  vi  EuropeSNPstart2reverse.pl
2098  top
2099  ls -l *reverse.pl
2100  ls -l Pop*
2101  vi  PopulationSpecific1000gCHIreverse.pl
2102  ls -l *reverse.pl
2103  perl ChinaSNPstart2reverse.pl
2104  top
2105  history
```

April 10, 2022

Continent-specific Allele Fixation
EXAMPLE:

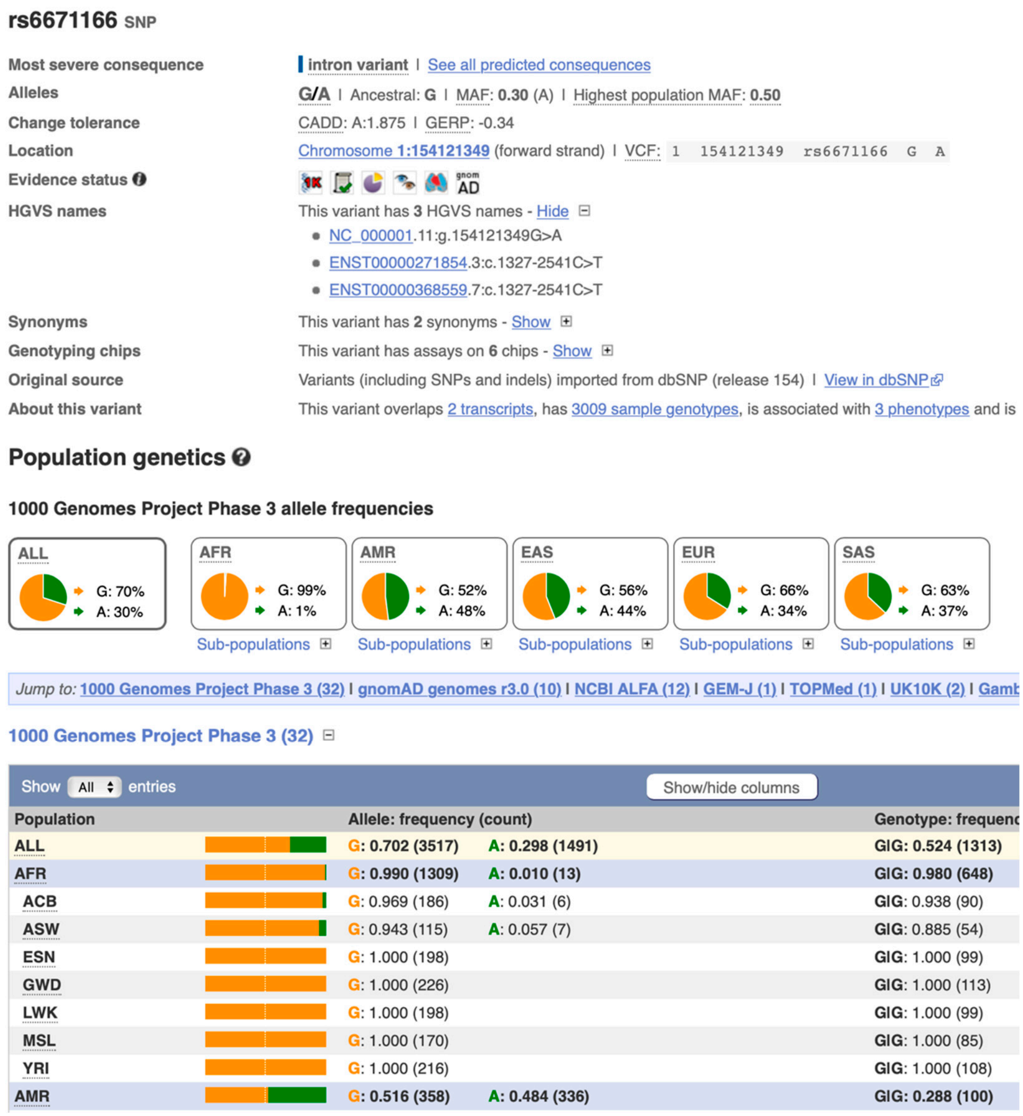

rs6671166 SNP

Most severe consequence	intron variant | See all predicted consequences
Alleles	G/A | Ancestral: G | MAF: 0.30 (A) | Highest population MAF: 0.50
Change tolerance	CADD: A:1.875 | GERP: -0.34
Location	Chromosome 1:154121349 (forward strand) | VCF: 1 154121349 rs6671166 G A
Evidence status	
HGVS names	This variant has 3 HGVS names - Hide<br>• NC_000001.11:g.154121349G>A<br>• ENST00000271854.3:c.1327-2541C>T<br>• ENST00000368559.7:c.1327-2541C>T
Synonyms	This variant has 2 synonyms - Show
Genotyping chips	This variant has assays on 6 chips - Show
Original source	Variants (including SNPs and indels) imported from dbSNP (release 154) | View in dbSNP
About this variant	This variant overlaps 2 transcripts, has 3009 sample genotypes, is associated with 3 phenotypes and is

Population genetics

1000 Genomes Project Phase 3 allele frequencies

ALL	AFR	AMR	EAS	EUR	SAS
G: 70%<br>A: 30%	G: 99%<br>A: 1%	G: 52%<br>A: 48%	G: 56%<br>A: 44%	G: 66%<br>A: 34%	G: 63%<br>A: 37%
	Sub-populations	Sub-populations	Sub-populations	Sub-populations	Sub-populations

Jump to: 1000 Genomes Project Phase 3 (32) | gnomAD genomes r3.0 (10) | NCBI ALFA (12) | GEM-J (1) | TOPMed (1) | UK10K (2) | Gamb

1000 Genomes Project Phase 3 (32)

Show All entries   Show/hide columns

Population	Allele: frequency (count)		Genotype: frequenc
ALL	G: 0.702 (3517)	A: 0.298 (1491)	G|G: 0.524 (1313)
AFR	G: 0.990 (1309)	A: 0.010 (13)	G|G: 0.980 (648)
ACB	G: 0.969 (186)	A: 0.031 (6)	G|G: 0.938 (90)
ASW	G: 0.943 (115)	A: 0.057 (7)	G|G: 0.885 (54)
ESN	G: 1.000 (198)		G|G: 1.000 (99)
GWD	G: 1.000 (226)		G|G: 1.000 (113)
LWK	G: 1.000 (198)		G|G: 1.000 (99)
MSL	G: 1.000 (170)		G|G: 1.000 (85)
YRI	G: 1.000 (216)		G|G: 1.000 (108)
AMR	G: 0.516 (358)	A: 0.484 (336)	G|G: 0.288 (100)

The data are in the files on BIO server:

```
-rw-rw-r-- 1 afedorov afedorov    316807 апр 10 18:23 PopulationSpecificSNPsAFR1000reverse_ALL
-rw-rw-r-- 1 afedorov afedorov    515400 апр 10 18:23 PopulationSpecificSNPsCHI1000reverse_ALL
-rw-rw-r-- 1 afedorov afedorov      2598 апр 10 18:21 PopulationSpecificSNPsEUR1000reverse_ALL
```

Also they are transformed into Excel tables:

```
-rw-r--r--@ 1 afedorov  staff   430286 Apr 10 14:04 FixedAllelesAFR.xlsx
-rw-r--r--@ 1 afedorov  staff   674057 Apr 10 14:06 FixedAllelesEAS.xlsx
-rw-r--r--@ 1 afedorov  staff    13867 Apr 10 14:09 FixedAllelesEUR.xlsx
Alexeis-iMac:ATLAS3sept2020 afedorov$
```

For example FixedAllelesEUR.xlsx:
In the last 6 columns is number of allele counts in 5 continents. Counts of EUR in this table is <10.

chromosome	Position	RS-ids	#alleles AFR	#alleles AMR	#alleles EAS	#alleles EUR	#alleles SAS	#alleles TOTAL
1	31620274	rs533645834	969	50	126	5	59	1083
1	45778640	rs112417297	950	54	183	9	18	1031
2	39875246	rs72934232	967	60	72	2	66	1095
2	40533600	rs61681991	937	72	74	8	18	1035
2	200389045	rs544995569;rs111806968	1096	63	18	6	28	1193
3	75226426	rs538744842;rs113505457	723	180	153	3	126	1032
3	156039668	rs6773597	870	44	54	6	186	1106
3	188113131	rs869631	882	54	14	4	286	1226
4	9413414	rs10009417	904	57	140	2	162	1125
4	34522861	rs543621960;rs112657587	1047	74	22	5	48	1174
4	190831703	rs2338002	1030	74	230	2	18	1124
5	174485090	rs1875965	992	80	82	8	16	1096
6	102606964	rs561936033;rs564433224	952	38	12	6	62	1058
6	141880662	rs528476876	946	119	14	6	37	1108
8	35797290	rs6986608	785	63	185	5	162	1015
8	35803681	rs6987006	785	63	191	5	162	1015
8	106901957	rs7001221	962	92	18	0	54	1108
8	116765116	rs55932928	802	128	36	7	70	1007
8	116786471	rs73706214	795	158	84	8	62	1023
9	21933125	rs2188126	829	40	54	7	126	1002
9	21936381	rs7875199	835	40	54	7	126	1008
9	36745892	rs11998844	796	115	204	8	284	1203
9	89541597	rs149736480	1057	179	47	8	11	1255
9	100436598	rs559260094;rs11300168	893	229	305	7	52	1181
9	100440034	rs530688400;rs530688400;rs530688400;rs533050515	822	132	155	8	61	1023
9	117107340	rs553726877;rs143632430	963	61	107	1	87	1112
11	22281515	rs7124796	911	62	446	8	162	1143
12	46895231	rs560933247;rs552960140	995	316	162	4	61	1376
12	132844471	rs374414835	897	32	118	3	143	1075
13	33428261	rs550256702;rs200565222	908	57	11	6	61	1032
15	29152317	rs143431483	806	56	11	3	149	1014
15	45701904	rs532073340;rs2453545	1114	71	16	8	136	1329

15	48392165	rs1834640	954	218	931	6	308	1486	
15	48394586	rs57108441	749	210	929	7	254	1220	
15	48396808	rs1559857	698	132	493	4	212	1046	
15	48400199	rs2675345	952	233	763	5	268	1458	
15	48411821	rs2675346	698	132	496	1	210	1041	
15	48414969	rs2433354	696	133	494	1	208	1038	
15	48418645	rs2675347	696	134	494	6	211	1047	
15	48420744	rs2675348	712	134	494	1	211	1058	
15	48426484	rs1426654	953	285	996	3	308	1549	
15	48433494	rs2470102	952	235	759	6	266	1459	
15	48438269	rs2469597	694	132	492	2	211	1039	
15	48444748	rs2459394	694	134	493	6	213	1047	
15	48445387	rs2675349	694	134	494	1	212	1041	
15	101407442	rs11247239	1417	247	93	8	69	1741	
16	12747232	rs538270522;rs373765644;rs150214949	959	39	19	1	30	1029	
17	59070441	rs7214111	1341	70	186	4	14	1429	
18	11353065	rs9966812	908	46	118	4	270	1228	
18	32916594	rs2974909	982	37	17	4	26	1049	
19	42392130	rs28396197	851	52	185	8	176	1087	
20	19286867	rs6035287	809	199	281	8	62	1078	
21	17877794	rs2823850	877	70	61	8	98	1053	
21	17917666	rs28647658	861	76	67	8	101	1046	
21	17928554	rs2823880	877	75	77	8	106	1066	

NOTES:

1) It is better to normalize number of counts for each table in order to take into account admixtures. For example, for Europe, there is large admixture with AMR, SAS
2) I calculated only 3 tables for AFR, EAS, and EUR. The computation was done based on 1000Genomes, thus, OCE is missing.
3) Surprisingly, The Europe has the list number of fixed continent-specific alleles. East Asia has >10x more.

Below is the most prominent case for Europe:

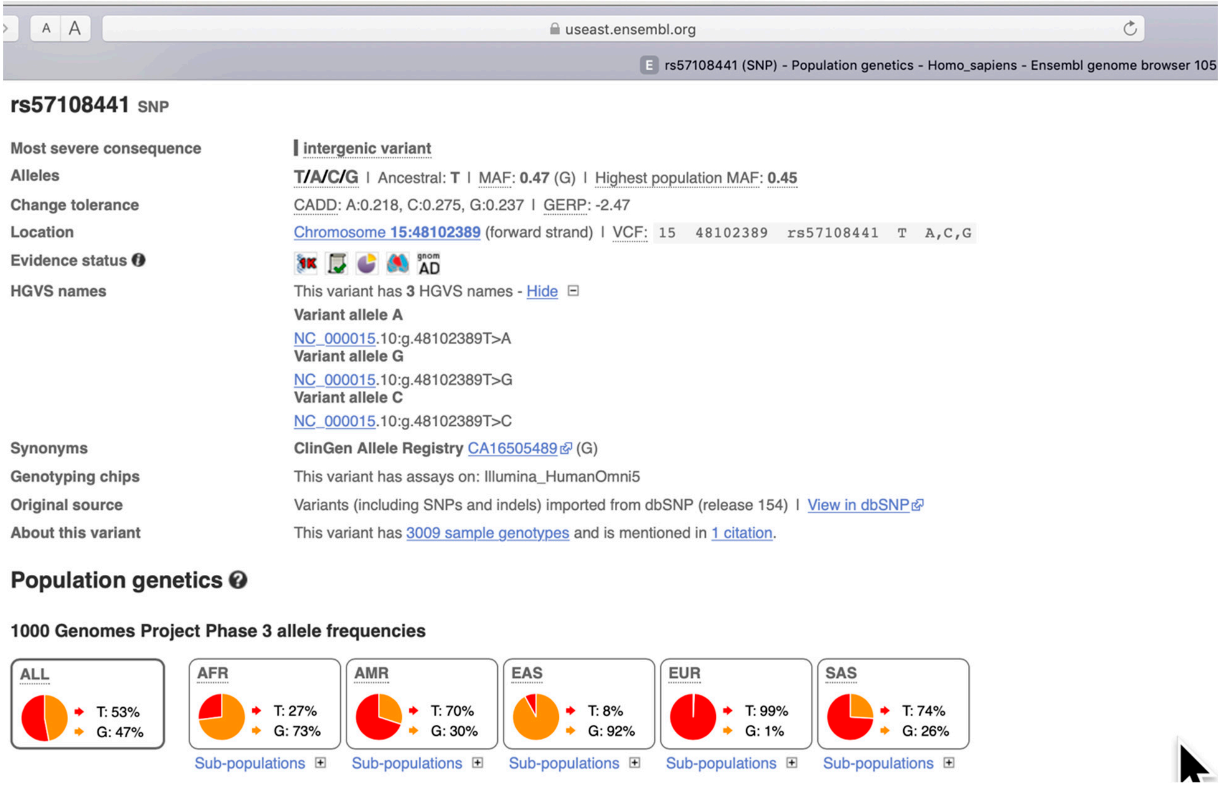

Computation of the reverse case (0) instead of (1) for fixation:

HISTORY:

```
 2023  cp PopulationSpecific1000gEURreverse.pl PopulationSpecific1000gEURreverseCOMP.pl
 2024  vi PopulationSpecific1000gEURreverseCOMP.pl
 2025  history 60
 2026  ls -l *pl
 2027  vi EuropeSNPstart2reverse.pl
 2028  perl EuropeSNPstart2reverse.pl
```

Figures for PaperAtlas3

rs34137121 SNP

**Most severe consequence**	**intron variant** I See all predicted consequences
**Alleles**	**C/G** I Ancestral: **C** I MAF: **0.18** (G) I Highest population MAF: **0.48**
**Change tolerance**	CADD: G:4.456 I GERP: 0.28
**Location**	Chromosome **15:39371864** (forward strand) I VCF: `15 39371864 rs34137121 C G`
**Evidence status**	1K AD
**HGVS names**	This variant has **4** HGVS names - Hide<br>• NC_000015.10:g.39371864C>G<br>• ENST00000558209.1:n.99-11723G>C<br>• ENST00000559318.1:n.408+48680G>C<br>• ENST00000560484.1:n.67+49045G>C
**Synonyms**	**ClinGen Allele Registry** CA14077454 (G)
**Original source**	Variants (including SNPs and indels) imported from dbSNP (release 154) I View in dbSNP
**About this variant**	This variant overlaps 3 transcripts and has 3009 sample genotypes.

Population genetics

1000 Genomes Project Phase 3 allele frequencies

ALL	AFR	AMR	EAS	EUR	SAS
C: 82%	C: 98%	C: 75%	C: 99%	C: 52%	C: 79%
G: 18%	G: 2%	G: 25%	G: 1%	G: 48%	G: 21%
	Sub-populations	Sub-populations	Sub-populations	Sub-populations	Sub-populations

Jump to: 1000 Genomes Project Phase 3 (32) I gnomAD genomes r3.0 (10) I NCBI ALFA (12) I GEM-J (1) I TOPMed (1) I UK10K (2) I Gambia

1000 Genomes Project Phase 3 (32)

Show All entries Show/hide columns

Population	Allele: frequency (count)		Genotype: frequency (count)	
**ALL**	**C: 0.823 (4120)**	**G: 0.177 (888)**	**C|C: 0.707 (1770)**	**C|G: 0.232 (580)**
**AFR**	**C: 0.983 (1300)**	**G: 0.017 (22)**	**C|C: 0.968 (640)**	**C|G: 0.030 (20)**
ACB	C: 0.958 (184)	G: 0.042 (8)	C|C: 0.917 (88)	C|G: 0.083 (8)
ASW	C: 0.910 (111)	G: 0.090 (11)	C|C: 0.836 (51)	C|G: 0.148 (9)
ESN	C: 1.000 (198)		C|C: 1.000 (99)	

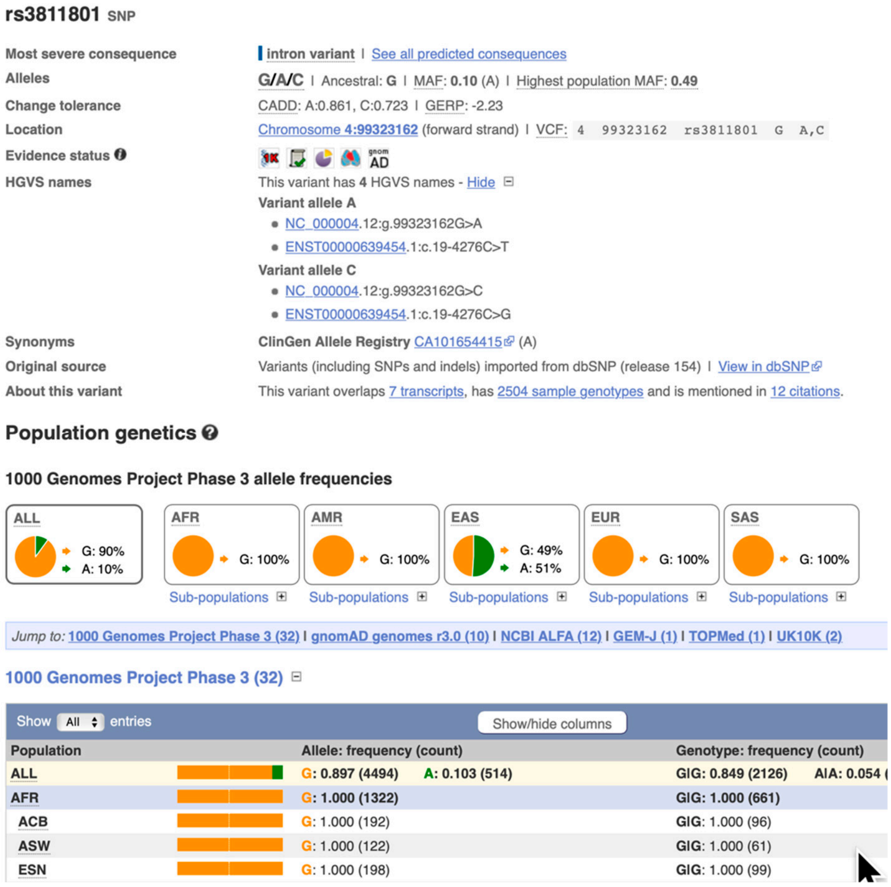
rs3811801 SNP
Most severe consequence
intron variant | See all predicted consequences
Alleles
G/A/C | Ancestral: G | MAF: 0.10 (A) | Highest population MAF: 0.49
Change tolerance
CADD: A:0.861, C:0.723 | GERP: -2.23
Location
Chromosome 4:99323162 (forward strand) | VCF: 4 99323162 rs3811801 G A,C
Evidence status
HGVS names
This variant has 4 HGVS names - Hide
Variant allele A
NC_000004.12:g.99323162G>A
ENST00000639454.1:c.19-4276C>T
Variant allele C
NC_000004.12:g.99323162G>C
ENST00000639454.1:c.19-4276C>G
Synonyms
ClinGen Allele Registry CA101654415 (A)
Original source
Variants (including SNPs and indels) imported from dbSNP (release 154) | View in dbSNP
About this variant
This variant overlaps 7 transcripts, has 2504 sample genotypes and is mentioned in 12 citations.
Population genetics
1000 Genomes Project Phase 3 allele frequencies
ALL G: 90% A: 10%
AFR G: 100%
AMR G: 100%
EAS G: 49% A: 51%
EUR G: 100%
SAS G: 100%
Sub-populations
Jump to: 1000 Genomes Project Phase 3 (32) | gnomAD genomes r3.0 (10) | NCBI ALFA (12) | GEM-J (1) | TOPMed (1) | UK10K (2)
1000 Genomes Project Phase 3 (32)
Show All entries
Show/hide columns
Population
Allele: frequency (count)
Genotype: frequency (count)
ALL G: 0.897 (4494) A: 0.103 (514) G|G: 0.849 (2126) A|A: 0.054
AFR G: 1.000 (1322) G|G: 1.000 (661)
ACB G: 1.000 (192) G|G: 1.000 (96)
ASW G: 1.000 (122) G|G: 1.000 (61)
ESN G: 1.000 (198) G|G: 1.000 (99)

rs7654389 SNP

**Most severe consequence**	**intron variant** I See all predicted consequences
**Alleles**	**C/A/G/T** I Ancestral: **T** I MAF: **0.15** (T) I Highest population MAF: **0.50**
**Change tolerance**	CADD: A:0.982, G:1.066, T:1.304 I GERP: -0.43
**Location**	Chromosome **4:84193752** (forward strand) I VCF: `4 84193752 rs7654389 C A,G,T`
**Evidence status**	gnom AD
**HGVS names**	This variant has **15** HGVS names - Hide

Variant allele T

- NC_000004.12:g.84193752C>T
- ENST00000508406.1:n.580-44496G>A
- ENST00000513489.5:n.228+49359G>A
- ENST00000657959.1:n.156-44496G>A
- ENST00000668493.1:n.643-45622G>A

Variant allele A

- NC_000004.12:g.84193752C>A
- ENST00000508406.1:n.580-44496G>T
- ENST00000513489.5:n.228+49359G>T
- ENST00000657959.1:n.156-44496G>T
- ENST00000668493.1:n.643-45622G>T

Variant allele G

- NC_000004.12:g.84193752C>G
- ENST00000508406.1:n.580-44496G>C
- ENST00000513489.5:n.228+49359G>C
- ENST00000657959.1:n.156-44496G>C
- ENST00000668493.1:n.643-45622G>C

**Synonyms**	This variant has **4** synonyms - Show
**Original source**	Variants (including SNPs and indels) imported from dbSNP (release 154) I View in dbSNP
**About this variant**	This variant overlaps 4 transcripts and has 3009 sample genotypes.

Population genetics

1000 Genomes Project Phase 3 allele frequencies

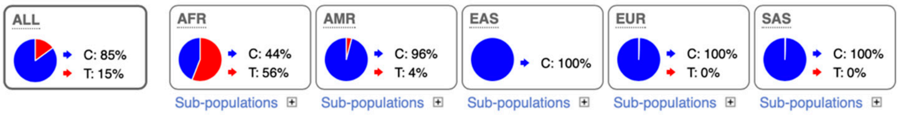

Jump to: **1000 Genomes Project Phase 3 (32)** I **gnomAD genomes r3.0 (10)** I **NCBI ALFA (12)** I **TOPMed (1)** I **UK10K (2)** I **Gambian Genome Va**

1000 Genomes Project Phase 3 (32)

Show All entries   Show/hide columns

Population	Allele: frequency (count)		Genotype: frequency (c	
**ALL**	**C: 0.845 (4234)**	**T: 0.155 (774)**	**C|C: 0.776 (1942)**	**C|**
**AFR**	**C: 0.436 (576)**	**T: 0.564 (746)**	**C|C: 0.189 (125)**	**C|**
**ACB**	**C**: 0.500 (96)	**T**: 0.500 (96)	**C|C**: 0.281 (27)	**C|**
**ASW**	**C**: 0.467 (57)	**T**: 0.533 (65)	**C|C**: 0.197 (12)	**C|**
**ESN**	**C**: 0.404 (80)	**T**: 0.596 (118)	**C|C**: 0.182 (18)	**C|**
**GWD**	**C**: 0.451 (102)	**T**: 0.549 (124)	**C|C**: 0.195 (22)	**C|**
**LWK**	**C**: 0.384 (76)	**T**: 0.616 (122)	**C|C**: 0.141 (14)	**C|**
**MSL**	**C**: 0.418 (71)	**T**: 0.582 (99)	**C|C**: 0.153 (13)	**C|**
**YRI**	**C**: 0.435 (94)	**T**: 0.565 (122)	**C|C**: 0.176 (19)	**C|**

```
afedorov@bio:~/NOV2021$ more PopulationSpecificSNPsEUR1000reverseCOMP_ALL
5       165608363      rs830694      796     162     191     1       54      1013
7       102726056      rs4727559     617     70      12      7       363     1057
11      113869356      rs7930014     806     165     166     9       128     1108
13      25906675       rs114576175;rs3839990  745     129     204     6       154     1034
14      69314540       rs386768      504     77      20      7       512     1100
14      77353103       rs7157792     811     149     253     8       75      1043
15      48419386       rs2555364     675     132     492     1       211     1019
15      48448864       rs8037198     695     131     492     1       211     1038
15      48460188       rs8028919     794     136     493     1       212     1143
15      48461146       rs3817315     695     131     493     1       212     1039
15      48471615       rs11070628    739     63      438     0       229     1031
15      48485926       rs2413887     951     284     990     3       296     1534
```

```
15    48506574    rs8023477   741   62    429   0     201   1004
15    48514309    rs9920281   780   140   490   6     219   1145
17    60529690    rs4728116   899   160   427   7     57    1123
17    60531329    rs7218749   899   160   426   7     56    1122
afedorov@bio:~/NOV2021$ history 30
 2027  vi EuropeSNPstart2reverse.pl
 2028  perl EuropeSNPstart2reverse.pl
 2029  history
 2030  ls
 2031  ls -l *ALL
 2032  more  FinalAfrica_ALL
 2033  sort -k2 -rn  FinalAfrica_ALL |more
 2034  ls -l *ALL
 2035  more FinalEastAsia_ALL
 2036  less -S FinalEastAsia_ALL
 2037  sort -k2 -rn  FinalEastAsia_ALL |less -S
 2038  ls -l *ALL
 2039  less -S  FinalEurope_ALL
 2040  sort -k2 -nr  FinalEurope_ALL |less -S
 2041  top
 2042  ls -l Pop*
 2043  top
 2044  ls -l Pop*
 2045  top
 2046  ls -l Pop*
 2047  more  PopulationSpecificSNPsEUR1000reverseCOMP_17
 2048  top
 2049  ls -l Pop*
 2050  more  PopulationSpecificSNPsEUR1000reverseCOMP_15
 2051  more PopulationSpecificSNPsEUR1000reverse_15
 2052  ls -l Pop*
 2053  perl ConcatenateFiles.pl PopulationSpecificSNPsEUR1000reverseCOMP_
 2054  ls -l Pop*
 2055  more PopulationSpecificSNPsEUR1000reverseCOMP_ALL
 2056  history 30
```

I checked a reciprocal case (0|1), when changed 1 to 0 in PopulationSpecific1000gEURreverseCOMP.pl and computed PopulationSpecificSNPsEUR1000reverseCOMP_17 files for Europe. Got 16 cases only (see page above).

April 11, 2022

I got a lot of triple alleles in ‘REVERSE’ computations:

```
13    25906675    rs114576175;rs3839990   745   129   204   6     154   1034
```

I need to remove them from the Table 2.

April 15, 2022

GOAL: Calculation of ancestral alleles from the field “AA=g” in the column #8 of Human VCF 1000G. APPROACH: Creation of a short version of VCF 1000G with only columns #1, 2, 3, 4, 5, and 8. They are in the files: 8COLUMNSchr_1..22.   Done by a command line highlighted in yellow below.

```
zcat ALL.chr22.phase3_shapeit2_mvncall_integrated_v5a.20130502.genotypes.vcf.gz |cut -
f1,2,3,4,5,8
 2021  zcat ALL.chr22.phase3_shapeit2_mvncall_integrated_v5a.20130502.genotypes.vcf.gz |cut -
f1,2,3,4,5,8 >8COLUMNSchr_22
 2022  ls -l ~/NOV2021/*pl
 2023  ls -l ~/NOV2021/ConcatenateFiles.pl
```

```
 2024  more  ~/NOV2021/ConcatenateFiles.pl
 2025  cp  ~/NOV2021/ConcatenateFiles.pl .
 2026  ls
 2027  cp ConcatenateFiles.pl 8columns.pl
 2028  vi 8columns.pl
 2029  history 40
 2030  vi 8columns.pl
 2031  perl 8columns.pl
 2032  vi 8columns.pl
 2033  perl 8columns.pl
 2034  ls -l
 2035  rm 8columns.pl
 2036  more 8COLUMNSchr_1
 2037  ls
 2038  ls *pl
 2039  history
 2040  vi ancestral.pl
 2041  ls
 2042  vi ancestral.pl
 2043  perl ancestral.pl
 2044  more 8COLUMNSchr_11
 2045  vi ancestral.pl
 2046  perl ancestral.pl
 2047  vi ancestral.pl
 2048  perl ancestral.pl
 2049  vi ancestral.pl
 2050  perl ancestral.pl
 2051  vi ancestral.pl
 2052  perl ancestral.pl
 2053  vi ancestral.pl
 2054  perl ancestral.pl
 2055  vi ancestral.pl
 2056  perl ancestral.pl
 2057  vi ancestral.pl
 2058  perl ancestral.pl
 2059  vi ancestral.pl
 2060  perl ancestral.pl
 2061  vi ancestral.pl
 2062  perl ancestral.pl
 2063  vi ancestral.pl
 2064  perl ancestral.pl
 2065  history 50
afedorov@bio:~/2500GENOMES$ pwd
/home/afedorov/2500GENOMES
```

PROGRAM ancestral.pl:

```
#!/usr/local/perl
#chomp($ARGV[0]);
#$x =$ARGV[0];

for $x (1..22) {
$c=0;

print "Chromosome $x \n";
$name = '8COLUMNSchr_' . $x;
$out = 'AncestralChr_' . $x;
open(OUT, ">$out");
open(IN, "$name") or die "cannot open chr $x \n";
while(<IN>) {
        chomp;
        $c++;
        if($c<254) {next;}
        $aa=''; #ancestral allele
        $X='nnn'; #unsestral status
        $L1=$L2=0; #SNP length
        @line=split(/\t/, $_);
        $line[3]=~s/\s//g;
        $line[4]=~s/\s//g;
        if ($line[5] =~/AA=([agtcAGTC]+)/) {$aa=uc($1);    }
        if ($line[3] eq $aa) {$X='AA=ref';}
        elsif ($line[4] eq $aa) {$X='AA=alt';}
```

```
        else {$X='AA=nnn';}
        $L1=length($line[3]); $L2=length($line[4]);
        if($L1 !=1 || $L2!=1) {$X='INDEL';}
        if ($line[3] =~/,/ || $line[4] =~/,/) {$X='TRIPLT';  } #tri-allele SNP
        print OUT "$X\t$line[3]\t$line[4]\t$line[1]\t$line[2]\t$aa\n";
}
close(OUT); close(IN);
}
```

From email to ATLAS3 team:

Hi Gennady,
You are absolutely right, in the files I sent you there is a fraction that presents triple-allele SNPs. These cases are misleading,
therefore, I removed them. Now the cleaned files with only di-allele SNPs are in BIO server /home/afedorov/NOV2021
The names of these three tables are the following:
PopulationSpecificSNPsAFR1000reverse_ALL_2allele
PopulationSpecificSNPsCHI1000reverse_ALL_2allele
PopulationSpecificSNPsEUR1000reverse_ALL_2allele

These are final files for rsflSNP alleles from AFR, EUR, and EAS

The data for rscSNP alleles are in the files FinalRegion_ALL (all chromosomes)

```
afedorov@bio:~/NOV2021$ ls -l Final*ALL
-rw-rw-r-- 1 afedorov afedorov 6683791 апр  2 23:43 FinalAfrica_ALL
-rw-rw-r-- 1 afedorov afedorov  471472 апр  2 23:44 FinalAmerica_ALL
-rw-rw-r-- 1 afedorov afedorov  145722 апр  2 23:44 FinalEastAsia_ALL
-rw-rw-r-- 1 afedorov afedorov 1086531 апр  2 23:44 FinalEurope_ALL
-rw-rw-r-- 1 afedorov afedorov  196742 апр  2 23:41 FinalOceania_ALL
```

April 16, 2022

Created program AddAncestral.pl for adding Ancestral SNP status for rscSNPs.
Obtained new tables:

```
afedorov@bio:~/NOV2021$ ls -l *ALL_AA
-rw-rw-r-- 1 afedorov afedorov 4535233 апр 17 03:43 FinalAfrica_ALL_AA
-rw-rw-r-- 1 afedorov afedorov  190966 апр 17 03:43 FinalAmerica_ALL_AA
-rw-rw-r-- 1 afedorov afedorov   20904 апр 17 03:43 FinalEastAsia_ALL_AA
-rw-rw-r-- 1 afedorov afedorov  114153 апр 17 03:43 FinalEurope_ALL_AA
-rw-rw-r-- 1 afedorov afedorov   78017 апр 17 03:43 FinalOceania_ALL_AA
```

Obtained new tables
Now working on fixed/lost SNPs program: AddAncestral_rsflSNP.pl
Done!

```
afedorov@bio:~/NOV2021$ ls -l Pop*alle*
-rw-rw-r-- 1 afedorov afedorov 314824 апр 12 03:33
PopulationSpecificSNPsAFR1000reverse_ALL_2allele
```

```
-rw-rw-r-- 1 afedorov afedorov 392628 апр 17 04:34
PopulationSpecificSNPsAFR1000reverse_ALL_2allele_AA
-rw-rw-r-- 1 afedorov afedorov 498606 апр 12 03:32
PopulationSpecificSNPsCHI1000reverse_ALL_2allele
-rw-rw-r-- 1 afedorov afedorov 625653 апр 17 04:34
PopulationSpecificSNPsCHI1000reverse_ALL_2allele_AA
-rw-rw-r-- 1 afedorov afedorov   1937 апр 12 03:33
PopulationSpecificSNPsEUR1000reverse_ALL_2allele
-rw-rw-r-- 1 afedorov afedorov   2571 апр 17 04:34
PopulationSpecificSNPsEUR1000reverse_ALL_2allele_AA
```

Below is region-specific fixed/loss SNPs from Europe.
A majority are triplets! Must be removed.

```
afedorov@bio:~/NOV2021$ more  PopulationSpecificSNPsEUR1000reverse_ALL_2allele_AA
1      31620274    rs533645834 TRIPLT     G      GA,GAA,GAAA 969    50     126   5      59      1083
1      45778640    rs112417297 TRIPLT     C      CTTTTTTTC,CTTTTTTTT     950   54      183     9
       18     1031
2      39875246    rs72934232  TRIPLT     C      G,T    967    60     72     2      66      1095
2      40533600    rs61681991  TRIPLT     G      GA,GC,GGC    937    72     74     8      18      1035
3      156039668   rs6773597   TRIPLT     G      A,T    870    44     54     6      186     1106
3      188113131   rs869631    TRIPLT     C      A,T    882    54     14     4      286     1226
4      9413414     rs10009417  TRIPLT     G      A,T    904    57     140    2      162     1125
4      190831703   rs2338002   TRIPLT     C      G,T    1030   74     230    2      18      1124
5      174485090   rs1875965   TRIPLT     T      C,G    992    80     82     8      16      1096
6      141880662   rs528476876 TRIPLT     C      CCTAT,CCTATCTAT,CCTATCTATCTAT,CCTATCTATCTATCTAT
       946    119    14     6      37     1108
8      35797290    rs6986608   AA=alt     T      G      785    63     185    5      162     1015
8      35803681    rs6987006   AA=alt     A      G      785    63     191    5      162     1015
8      106901957   rs7001221   TRIPLT     T      C,G    962    92     18     0      54      1108
8      116765116   rs55932928  AA=alt     A      G      802    128    36     7      70      1007
8      116786471   rs73706214  AA=nnn     T      C      795    158    84     8      62      1023
9      21933125    rs2188126   AA=alt     G      C      829    40     54     7      126     1002
9      21936381    rs7875199   AA=alt     T      C      835    40     54     7      126     1008
9      36745892    rs11998844  AA=ref     A      G      796    115    204    8      284     1203
9      89541597    rs149736480 TRIPLT     A      ATTAT,ATTATTTAT,ATTATTTATTTATTTAT    1057    179
       47     8      11     1255
11     22281515    rs7124796   TRIPLT     T      A,G    911    62     446    8      162     1143
12     132844471   rs374414835 TRIPLT     T      TCA,TCACA    897    32     118   3      143     1075
15     29152317    rs143431483 AA=nnn     A      G      806    56     11     3      149     1014
15     48392165    rs1834640   AA=alt     A      G      954    218    931    6      308     1486
15     48394586    rs57108441  AA=ref     T      G      749    210    929    7      254     1220
15     48396808    rs1559857   AA=alt     G      A      698    132    493    4      212     1046
15     48400199    rs2675345   AA=alt     A      G      952    233    763    5      268     1458
15     48411821    rs2675346   AA=alt     C      T      698    132    496    1      210     1041
15     48414969    rs2433354   AA=alt     C      T      696    133    494    1      208     1038
15     48418645    rs2675347   AA=alt     A      G      696    134    494    6      211     1047
15     48420744    rs2675348   AA=alt     A      G      712    134    494    1      211     1058
15     48426484    rs1426654   AA=alt     A      G      953    285    996    3      308     1549
15     48433494    rs2470102   AA=alt     A      G      952    235    759    6      266     1459
15     48438269    rs2469597   AA=alt     C      T      694    132    492    2      211     1039
15     48444748    rs2459394   AA=alt     T      A      694    134    493    6      213     1047
15     48445387    rs2675349   AA=alt     A      G      694    134    494    1      212     1041
15     101407442   rs11247239  TRIPLT     G      A,C,T  1417   247    93     8      69      1741
17     59070441    rs7214111   TRIPLT     A      G,T    1341   70     186    4      14      1429
18     11353065    rs9966812   TRIPLT     T      A,G    908    46     118    4      270     1228
18     32916594    rs2974909   TRIPLT     A      G,T    982    37     17     4      26      1049
19     42392130    rs28396197  AA=alt     T      C      851    52     185    8      176     1087
20     19286867    rs6035287   TRIPLT     A      C,T    809    199    281    8      62      1078
21     17877794    rs2823850   AA=alt     C      T      877    70     61     8      98      1053
21     17917666    rs28647658  AA=alt     C      T      861    76     67     8      101     1046
21     17928554    rs2823880   AA=alt     A      C      877    75     77     8      106     1066
afedorov@bio:~/NOV2021$
```

CONCLUSIONS!
OK. I finished insertion of Ancestral status. Found TRIPLETS (TRIPLT) that is better to remove.

Copied programs and new tables into:
Alexeis-iMac:ATLAS3sept2020 afedorov$ pwd
/Users/afedorov/Documents/ATLAS3sept2020

Need to prepare Excel tables and send to Co-authors tomorrow

April 17, 2022

Copied the data for Table1 and Table2 from BIO into MACcri:

1) Removed all Triplets (TRIPLT) from Table2, rsflSNPs and saved into new filenames:

```
grep -v TRI PopulationSpecificSNPsAFR1000reverse_ALL_2allele_AA >Table2rsflSNPafrica
grep -v TRI PopulationSpecificSNPsCHI1000reverse_ALL_2allele_AA >Table2rsflSNPeastasia
grep -v TRI PopulationSpecificSNPsEUR1000reverse_ALL_2allele_AA >Table2rsflSNPeurope
```

2) Counted number of derived (alt) rsflSNPs and ancestral (ref) rsflSNPs:

```
Alexeis-iMac:ATLAS3sept2020 afedorov$ ls -l Tab*
-rw-r--r--@ 1 afedorov  staff   34424 Apr 13 21:02 Table1_Atlas3march2022.xlsx
-rw-r--r--  1 afedorov  staff  387634 Apr 17 14:36 Table2rsflSNPafrica
-rw-r--r--  1 afedorov  staff  589341 Apr 17 14:38 Table2rsflSNPeastasia
-rw-r--r--  1 afedorov  staff    1383 Apr 17 14:38 Table2rsflSNPeurope
Alexeis-iMac:ATLAS3sept2020 afedorov$ grep alt Table2rsflSNPeastasia |wc
    3473   41676  193185
Alexeis-iMac:ATLAS3sept2020 afedorov$ grep ref Table2rsflSNPeastasia |wc
    5719   68628  319177
Alexeis-iMac:ATLAS3sept2020 afedorov$ grep ref Table2rsflSNPeurope |wc
       2      24     113
Alexeis-iMac:ATLAS3sept2020 afedorov$ grep alt Table2rsflSNPeurope |wc
      21     252    1159
Alexeis-iMac:ATLAS3sept2020 afedorov$ grep ref Table2rsflSNPafrica |wc
    5939   71268  332693
Alexeis-iMac:ATLAS3sept2020 afedorov$ grep alt Table2rsflSNPafrica |wc
     168    2016    9385
```

The highest number of ancestral “lost” rsflSNPs alleles was in Europe (ancestral=91.3% vs derived=8.7%), then EAS (ancestral=37.8 % vs derived=62.2%), then Africa (ancestral=2.8% vs derived=97.2%)

Processed data for rscSNPs. They do NOT have Triplets (TRI)

```
Alexeis-iMac:ATLAS3sept2020 afedorov$ grep ref FinalAfrica_ALL_AA |wc
   59130  886950 3445906
Alexeis-iMac:ATLAS3sept2020 afedorov$ grep alt FinalAfrica_ALL_AA |wc
   16429  246435  957223
Alexeis-iMac:ATLAS3sept2020 afedorov$ grep ref FinalAmerica_ALL_AA |wc
    3257   48855  185764
Alexeis-iMac:ATLAS3sept2020 afedorov$ grep alt FinalAmerica_ALL_AA |wc
      27     405    1537
Alexeis-iMac:ATLAS3sept2020 afedorov$ grep ref  FinalEastAsia_ALL_AA |wc
     318    4770   18337
Alexeis-iMac:ATLAS3sept2020 afedorov$ grep alt  FinalEastAsia_ALL_AA |wc
      22     330    1293
Alexeis-iMac:ATLAS3sept2020 afedorov$ grep ref   FinalEurope_ALL_AA |wc
    1823   27345  108920
Alexeis-iMac:ATLAS3sept2020 afedorov$ grep alt   FinalEurope_ALL_AA |wc
      51     765    3019
Alexeis-iMac:ATLAS3sept2020 afedorov$ grep ref    FinalOceania_ALL_AA |wc
     269    4035   15226
Alexeis-iMac:ATLAS3sept2020 afedorov$ grep alt    FinalOceania_ALL_AA |wc
      11     165     620
```

The highest number of ancestral rscSNP alleles was in Africa (ancestral=21.7% vs derived=78.3%), then much less in other continents: EAS (ancestral=6.5% vs derived=93.5%), America (ancestral=0.8% vs derived=99.2%), Europe (ancestral=2.7% vs derived=97.3%), Oceania (ancestral=3.9% vs derived=96.1%)

Created **Table1_data_Apr17** that contains data on rscSNP alleles in AFR, AMR, EAS, EUR, OCE

Created **Table2_data_Apr17** that contains data on rsflSNP alleles in 3 regions AFR, EAS, EUR

These two tables should be Supplementary tables for the ATLAS-3 paper.

April 19, 2022

Clustering Table 2.
HISTORY:

```
mv FinalAfrica_ALL_AA FinalAfrica_ALL_AA.tx
  600  mv FinalAfrica_ALL_AA.tx FinalAfrica_ALL_AA.txt
  601  mv FinalAmerica_ALL_AA FinalAmerica_ALL_AA.txt
  602  mv FinalEastAsia_ALL_AA FinalEastAsia_ALL_AA.txt
  603  mv FinalEurope_ALL_AA FinalEurope_ALL_AA.txt
  604  mv FinalOceania_ALL_AA FinalOceania_ALL_AA.txt
  605  ls
  606  ls -l *allele_AA
  607  mv PopulationSpecificSNPsAFR1000reverse_ALL_2allele_AA
PopulationSpecificSNPsAFR1000reverse_ALL_2allele_AA.txt
  608  mv PopulationSpecificSNPsCHI1000reverse_ALL_2allele_AA
PopulationSpecificSNPsCHI1000reverse_ALL_2allele_AA.txt
  609  mv PopulationSpecificSNPsEUR1000reverse_ALL_2allele_AA
PopulationSpecificSNPsEUR1000reverse_ALL_2allele_AA.txt
  610  ls
  611  ls -l Table2rs*
  612  mv Table2rsflSNPafrica Table2rsflSNPafrica.txt
  613  mv Table2rsflSNPeastasia Table2rsflSNPeastasia.txt
  614  mv Table2rsflSNPeurope Table2rsflSNPeurope.txt
  615  wc Table2rsflSNPeurope.txt
  616  wc Table2rsflSNPeastasia.txt
  617  wc Table2rsflSNPafrica.txt
  618  ls
  619  vi ClustersSNP.pl
  620  more Table2rsflSNPafrica.txt
  621  ls
  622  vi ClustersSNP.pl
  623  perl ClustersSNP.pl Table2rsflSNPafrica.txt
  624  vi ClustersSNP.pl
  625  vi ClustersSNP.pl
  626  perl ClustersSNP.pl Table2rsflSNPafrica.txt
  627  vi ClustersSNP.pl
  628  perl ClustersSNP.pl Table2rsflSNPeastasia.txt
  629  perl ClustersSNP.pl Table2rsflSNPeurope.txt
  630  history
```

OK. I calculated numbers of rsflSNP clusters and put the new data into Table 2

April 22, 2022

Computed Top 10% of Africa-specific rscSNPs from 77,820 SNPs in Table 1 (column 3). New filename: `FinalAfrica_rscSNPs10percent.` These SNPs are sorted by their frequency in Africa

IN MAC CRI command line:

```
Alexeis-iMac:ATLAS3sept2020 afedorov$ sort -k11 -nr FinalAfrica_ALL_AA.txt |head -7782
>FinalAfrica_rscSNPs10percent
```

I need to send this file to Gennady
