# Supplementary material for: Analysis of Common SNPs across Continents Reveals Major Genomic Differences between Human Populations
Source: Genes (Basel). 2022 Aug 18;13(8):1472. doi: 10.3390/genes13081472 (PMC9408407; doi:10.3390/genes13081472)
Supplement: Supplementary file 1 [file genes-13-01472-s001.zip › genes-1766591-supplementary/InstructionManualATLAS3.pdf]

Instruction manual for ATLAS3 project

Example for getting ARSA SNPs for Africa

1) Command lines for *AfricaSimonStep1.pl.*

Type in a Linux command line: `perl  AfricaSimonStep1.pl 19`
Here, the argument ($ARGV[0]) is the number of chromosome. In this example it is chromosome-19. This program requires two support files: `simon_pops2_A2.txt` and `ids_simon`. In addition, in line=40 a user must specify the path to the Simons' database: `$name = '/home/afedorov/simon/simon_chr'.$L.'.gz'`. Here "$L" is a chromosome number. In our example $L=19. The output filename is `AFR_SNPs_19`.
In order to run this perl program simultaneously for all chromosomes use another program with a command line:
Type in a Linux command line: `perl AfricanSNPstart1.pl`

2) Command lines for *Africa1000gStep2_v3.pl*

Type in a Linux command line: `perl  Africa1000gStep2_v3.pl 19`
Here, the argument ($ARGV[0]) is the number of chromosome. In this example it is chromosome-19. This program requires two support files: `2504ids` and `igsr_samples.tsv`, and also the output from the previous program - `AFR_SNPs_19`. In addition, in line=44 a user must specify the path to the 1000 Genomes database:
$name = '/home/afedorov/2500GENOMES/ALL.chr'.$L.'.phase3_shapeit2_mvncall_integrated_v5a.20130502.genotypes.vcf.gz'; . Here "$L" is a chromosome number. In our example $L=19. The output filename is `AfricaSpecificV3_19`.
In order to run this perl program simultaneously for all chromosomes use another program with a command line:
Type in a Linux command line: `perl AfricaSNPstart2.pl`

3) Command lines for *AfricanSNPsESTONIA.pl.*

Type in a Linux command line: `perl  AfricanSNPsESTONIA.pl 19`
Here, the argument ($ARGV[0]) is the number of chromosome. In this example it is chromosome-19. This program requires one support file: `estonian_pops2_A2.txt`, and also the output from the previous program - `AfricaSpecificV3_19`. In addition, in line=20 a user must specify the path to the Estonian database:
$name = '/home/afedorov/EGDP_PaganiEtAl2016_Release_Build37_VCF/EGDP_PaganiEtAl2016_Release_Build37_Chr'.$L.'.vcf.gz'. Here "$L" is a chromosome number. In our example $L=19. The outputs are three files with filenames:
`AfricanSpecificSNPsEstonia_19, AfricanSpecificSNPsEstonia2_19, and AfricanSpecificSNPsEstonia3_19`
In order to run this perl program simultaneously for all chromosomes use another program with a command line:
Type in a Linux command line: `perl AfricaSNPstart3.pl`

For getting ARSA SNPs for America, East Asia, Europe, and Oceania follow the protocol for Africa using the programs, that are specific for your region:

American ARSA – 1) *AmericanSNPs2020.pl*; 2) *America1000gStep2_v2.pl*; 3) *AmericanSNPsESTONIA.pl.* For East Asia ARSA – 1) *ChinaSimonStep1.pl*; 2) *China1000gStep2_v2.pl*; 3) *ChinaEstoniaStep3.pl.* For European ARSA – 1) *EuropeSimonStep1.pl*; 2) *Europe1000gStep2_v2.pl*; 3) *EuropeEstoniaStep3.pl.* For obtaining Oceania ARSA we used the following programs: 1) *OceaniaSimonStep1.pl*; 2) *Oceania1000gStep2.pl*; 3) *OceanEstoniaStep3.pl.*

Example for getting RACA SNPs for Africa

1) Command lines for *PopulationSpecific1000gAFRreverse.pl*

Type in a Linux command line: `perl  PopulationSpecific1000gAFRreverse.pl 19`
Here, the argument ($ARGV[0]) is the number of chromosome. In this example it is chromosome-19. This program requires two support files: `2504ids` and `igsr_samples.tsv`. In addition, in line=28 a user must specify the path to the 1000 Genomes database:
$name = '/home/afedorov/2500GENOMES/ALL.chr'.$L.'.phase3_shapeit2_mvncall_integrated_v5a.20130502.genotypes.vcf.gz'; . Here "$L"

is a chromosome number. In our example $L=19.   The output filename is
`5PopulationSpecificSNPsAFR1000reverse_19`.
In order to run this perl program simultaneously for all chromosomes use another program with a command line:
Type in a Linux command line: `perl AfricaSNPstart2reverse.pl`

For getting RACA SNPs for East Asia and Europe follow the protocol for Africa using the programs, that are specific for your region:
for East Asia RACA SNPs - *PopulationSpecific1000gCHIreverse.pl*; for European RACA - *PopulationSpecific1000gEURreverse.pl*.

The details of command lines are shown in the electronic notebook for this project
`ATLAS3_notebook_protocols.docx`
